# Data Driven Mathematical Model of FOLFIRI Treatment for Colon Cancer

**DOI:** 10.3390/cancers13112632

**Published:** 2021-05-27

**Authors:** Aparajita Budithi, Sumeyye Su, Arkadz Kirshtein, Leili Shahriyari

**Affiliations:** 1 Department of Mathematics and Statistics, University of Massachusetts Amherst, Amherst, MA 01003, USA; abudithi@umass.edu (A.B.); sumeyyesu@umass.edu (S.S.); 2 Department of Mathematics, Tufts University, Medford, MA 02155, USA; Arkadz.Kirshtein@tufts.edu

**Keywords:** colon cancer, FOLFIRI treatment, data-driven mathematical model, precision medicine, immune variations, gene expression profiles, digital cytometry, 5-FU, leucovorin, irinotecan

## Abstract

**Simple Summary:**

Since the micro-environment of colonic tumors, including their immune structure would affect the response to treatments, we study the response of five groups of tumors clustered based on their immune patterns to a common colon cancer treatment. We develop a data driven mathematical model to investigate the behavior of key players in colonic tumors in each of these clusters in response to the FOLFIRI treatment. Although the model shows clear differences in the behavior of tumors in different clusters, it cannot suggest a unique optimal treatment strategy for each cluster. The results show that there is not much difference in the dynamics of tumors in response to 5-FU alone versus 5-FU plus Leucovorin. However, adding Irinotecan changes the dynamics of T-reg and dendritic cells leading to a remarkably slower tumor recurrence, especially for tumors in a cluster, which has the highest level of T-reg/T-helper ratio compared to the other clusters.

**Abstract:**

Many colon cancer patients show resistance to their treatments. Therefore, it is important to consider unique characteristic of each tumor to find the best treatment options for each patient. In this study, we develop a data driven mathematical model for interaction between the tumor microenvironment and FOLFIRI drug agents in colon cancer. Patients are divided into five distinct clusters based on their estimated immune cell fractions obtained from their primary tumors’ gene expression data. We then analyze the effects of drugs on cancer cells and immune cells in each group, and we observe different responses to the FOLFIRI drugs between patients in different immune groups. For instance, patients in cluster 3 with the highest T-reg/T-helper ratio respond better to the FOLFIRI treatment, while patients in cluster 2 with the lowest T-reg/T-helper ratio resist the treatment. Moreover, we use ROC curve to validate the model using the tumor status of the patients at their follow up, and the model predicts well for the earlier follow up days.

## 1. Introduction

Colorectal cancer (CRC), the third most common cancer diagnosed in both men and women in the United States excluding skin cancers, is estimated to cause about 52,980 deaths during 2021 [1]. Surgical resection, radiation therapy and systemic therapies that use medications such as chemotherapy, targeted therapy, and immunotherapy are the treatment options depending on several factors such as the type and stage of the disease, the molecular analysis of the tumor, possible side effects and overall health of the patients [2,3]. Early stage tumors can be curable with surgical resection while many patients with advance stage and metastatic CRC receive chemotherapy as a combination of treatment [4]. Although overall mortality rate of CRC patients has been decreasing for several decades, reduction rate slowed over the past decade (2008–2017) [5]. Survival rate remains poor for patients with metastatic CRC despite advances in the primary treatment of chemotherapy [6]. Predicting variability in response to treatments to increase survival rate and arrive at precision medicine, we need to understand disease progression and determine the major drivers for each patient.

One of the major players in response to cancer treatments is immune system. Immune cells in the tumor microenvironment contact with tumor cells directly or through chemokine and cytokine signaling and they play essential roles in improvement and blocking of therapeutic efficacy and the behavior of the tumor [7]. Targeting tumor cells by radiotherapy and chemotherapy causes the release of damage-associated molecular pattern (DAMP) molecules such as high mobility group box 1 (HMGB1) as a result of necrotic cell death [8,9,10,11], and it has been found that HMGB1 triggers immune responses [12,13]. Activated CD8${}^{+}$ T-cells release a high level of cytokines such as IFN-γ and FasL that boost production of necrotic cells in colon cancer [14]. Dendritic cells are activated by HMGB1 that can be released from macrophages [10] and activated dendritic cells, and they cause activation of T-cells [15]. Moreover, tumor-associated macrophages (TAMs) are known as key regulators of therapeutic response in the tumor microenvironment. CD4${}^{+}$ T-cells release IFN-γ that activates $M_{1}$ macrophages [16,17], and CD4${}^{+}$ T-cells are activated by TNF-α, which is released by $M_{1}$ macrophages [18]. In contrast, M2 macrophages are activated by exposure to certain cytokines such as IL-4, IL-10 and IL-13 and elevate tumorigenesis [19,20].

Many studies have reported a relationship between clinical outcome and immune cells in colon cancer. For example, a high proportion of CD8+ T-cells, effector memory T-cells and CD4+ T-cells is correlated with longer survival in colorectal cancer [21,22,23]. Furthermore, it has been observed that radiotherapy mediates tumor regression because of the release of IFN-γ by CD8${}^{+}$ T-cells [24,25]. In addition, patients with high expression levels of the Th17 markers show a poor prognosis, while a high expression of the Th1 markers is associated with prolonged disease-free survival in colorectal cancer [26]. Moreover, it has been shown that TAMs mediate resistance to some chemotherapeutic agents such as 5-fluorouracil, doxorubicin [27].

Most chemotherapy treatments for colon cancer include Fluorouracil (5-FU), which is a fluoropyrimidine antimetabolite drug used for different cancers types such as colorectal, breast, head and neck cancers [28]. However, response rate of 5-FU-based chemotherapy as a first-line treatment for advanced colorectal cancer remains only 10–15% [29,30]. To overcome this therapeutic resistance, combinations of chemotherapy drugs such as FOLFIRI (Folinic acid, Fluorouracil and Irinotecan) are used for targeting tumor cells and the tumor microenvirinment simultaneously, and they have improved the response rates up to 40–50% for advanced colorectal cancer [29,31]. Accumulating evidence indicates that the relative abundance of various immune cells and their interaction network with treatment approaches are essential in the colonic tumors’ initiation and progression. Therefore, this study focuses on the interaction between tumor-infiltrating immune cells and FOLFIRI agents by dividing patients into similar cohorts based on their tumor-infiltrating immune variations to model the response to the cancer treatment.

Mathematical models are used in many studies to understand tumor growth dynamics, improve therapeutic responses, find the best treatment combination and overcome drug resistance in different cancer treatments [32,33,34,35,36,37,38,39,40]. The effect of radiotherapy and chemotherapy on tumor growth has been modelled using partial differential equations (PDEs), ordinary differential equations (ODEs) and linear quadratic models in breast and brain tumors [41,42,43]. Immune cell interactions with tumor cells are used as an alternative approach for the mathematical modeling of the cancer treatments in a few studies that pharmacokinetic ODEs are defined to predict the optimal dosing regimen and the combined effect of chemotherapy and immunotherapy [44,45,46,47]. Many of these models such as [45] can not be verified because of lack of time course data for the growth of treated and/or untreated tumors. For this reason, some models such as [46] have simulated outcomes for groups of virtual patients on treatment protocols for which clinical trial data are available, using a range of biologically reasonable patient-specific parameter values.

We have recently developed a data driven mathematical model of colon cancer with a focus on the key players and the interaction network between immune cells and cancer cells in order to discover differences in tumor growths of patients with different immune profiles [48]. In this study, we found that there are five distinct groups of primary colon tumors based on their immune profiles, which have been estimated from their gene expression profiles. To analyze the model’s predictions for tumors in each cluster, patient-specific parameters have been estimated using the data of each cluster [48]. In this study, we extend our previous model including the interaction between Fluorouracil, Leucovorin, Irinotecan, and various cell types in tumor to investigate the effect of these drugs on tumors in each cluster.

## 2. Materials & Methods

### 2.1. Mathematical Model

**Figure 1:**
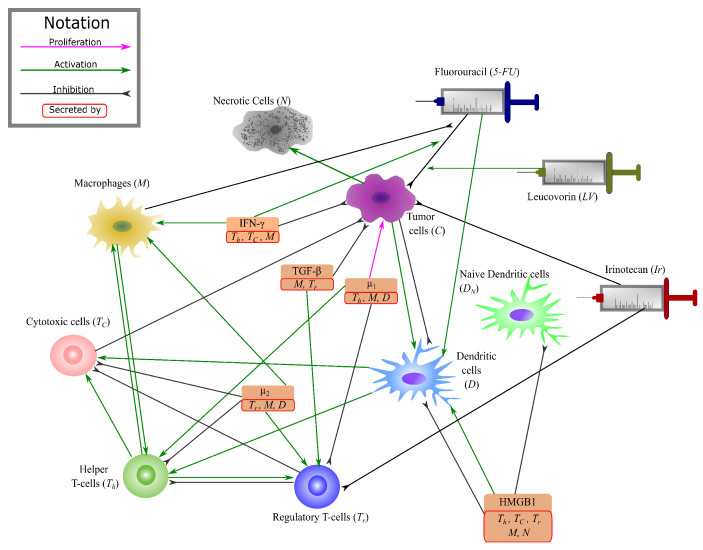
Interaction network of FOLFIRI drugs. Interaction network among the key players in tumor microenvironment and FOLFIRI drugs.

There is a complex web of numerous interactions in the colon cancer microenvironment. To be able to analyse and study the role of key interactions in tumors’ progression, the network of these interactions has been reduced to a clear and compact model in [48], highlighting the key components. This network model contains 14 variables that can be majorly grouped into T-cells, dendritic cells, macrophages, cytokines, cancer cells and necrotic cells. In this paper, we add the interactions of three FOLFIRI agents to this network to study the effect of these drugs on colonic tumors (Figure 1 and Table 1).

The interaction network among some of the key players in colon cancer as modeled in [48] is summarized below. In the model described in [48], individually modelled cytokines include HMGB1 (H), IFN-γ ($I_{\gamma }$), and TGF-β ($G_{\beta }$). The group of carcinogenic cytokines including IL-6, IL-17, IL-21 and IL-22 is modeled as one variable $\mu_{1}$, while the combined sum of immunosuppressive molecules, including IL-10 and CCL20 is modeled as $\mu_{2}$. $\mu_{1}$ is collectively modelled as secreted by macrophages [49,50,51,52], helper T-cells [51,52,53,54] and a sub-population of dendritic cells [55,56], and $\mu_{2}$ as produced by macrophages [57,58,59], dendritic cells [55,60] and T-reg cells [53,61,62,63]. HMGB1 is modeled as passively released from necrotic cells [64], or actively secreted from activated T-cells, and macrophages [65,66]. IFN-γ is secreted by a sub-population of macrophages [57,67,68,69,70], helper T-cells [16,17] and cytotoxic cells [14]. TGF-β is produced by macrophages [57,58] and T-reg cells [53,61,62,71].

As it has been described in [48], cells that have been modeled are T-cells, macrophages, dendritic cells, necrotic cells, and cancer cells. Naive T-cells ($T_{N}$) are included in the system to make the system more stable by modeling the activation rates of sub-types of T-cells proportional to the density of naive T-cells. Helper T-cells ($T_{h}$) are modeled as they are activated by dendritic cells or certain cytokines including IL-12, IL-6, and IL-23 [61]. Cytotoxic cells ($T_{C}$) are activated by IL-12, IL-4, IL-5 and IL-13 [15,72,73], and regulatory T-cells ($T_{r}$) are also shown to be activated by the cytokines IL-2 [61,74,75], CCL20 [59], and TGF-β [61,71]. Additionally, T-reg cells inhibit both $T_{h}$ and $T_{C}$ cells by various means, including the production of immunosuppresive cytokines [61]. The major effects on dendritic cells are by HMGB1, which activates [10] them but also reduce their maturation rate as shown by some sources [63,76]; and cancer cells, which indirectly may promote their apoptosis [76,77,78,79,80]. Dendritic cells are modelled as two types of naive ($D_{N}$) and activated (*D*). Macrophages are either activated by IFN-γ or by interleukins (ILs) [57,81,82]. Proliferation in cancer is taken to be proportional to $\left[C\right](1-\left[C\right]/C_{0})$, where $C_{0}$ [83,84] is the total capacity, with additional proliferation by cytokines [61,85], and IL-6 may cause an additional loss of apoptosis in cancer cells [77,79,86,87]. While, cancer development is suppressed by TGF-β, IL-12, IFN-γ and cytotoxic T-cells [61,88,89,90,91]. Here, necrotic cells are considered to be all those cells that go through the process of necrotic cell death and are modelled with a rate of *production* given by a fraction of dying cancer cells. This is because they are produced either naturally by the tumor, or due to the indirect effect of cytotoxic T-cells [14].

The ODE system obtained as a result of these interactions (presented in [48]) is:

(1) $$\begin{array}{cc} \frac{d\left[T_{N}\right]}{dt}= & A_{T_{N}}-\left(\lambda_{T_{h}D}\left[D\right]+\lambda_{T_{h}M}\left[M\right]+\lambda_{T_{h}\mu_{1}}\left[\mu_{1}\right]\right)\left[T_{N}\right]-\left(\lambda_{T_{C}T_{h}}\left[T_{h}\right]+\lambda_{T_{C}D}\left[D\right]\right)\left[T_{N}\right]\\ \; & -\left(\lambda_{T_{r}T_{h}}\left[T_{h}\right]+\lambda_{T_{r}\mu_{2}}\left[\mu_{2}\right]+\lambda_{T_{r}G_{\beta }}\left[G_{\beta }\right]\right)\left[T_{N}\right]-\delta_{T_{N}}\left[T_{N}\right] \end{array}$$

(2) $$\begin{array}{cc} \frac{d\left[T_{h}\right]}{dt}= & \left(\lambda_{T_{h}D}\left[D\right]+\lambda_{T_{h}M}\left[M\right]+\lambda_{T_{h}\mu_{1}}\left[\mu_{1}\right]\right)\left[T_{N}\right]-\left(\delta_{T_{h}\mu_{2}}\left[\mu_{2}\right]+\delta_{T_{h}T_{r}}\left[T_{r}\right]+\delta_{T_{h}}\right)\left[T_{h}\right] \end{array}$$

(3) $$\begin{array}{cc} \frac{d\left[T_{C}\right]}{dt}= & \left(\lambda_{T_{C}T_{h}}\left[T_{h}\right]+\lambda_{T_{C}D}\left[D\right]\right)\left[T_{N}\right]-\left(\delta_{T_{C}\mu_{2}}\left[\mu_{2}\right]+\delta_{T_{C}T_{r}}\left[T_{r}\right]+\delta_{T_{C}}\right)\left[T_{C}\right] \end{array}$$

(4) $$\begin{array}{cc} \frac{d\left[T_{r}\right]}{dt}= & \left(\lambda_{T_{r}T_{h}}\left[T_{h}\right]+\lambda_{T_{r}\mu_{2}}\left[\mu_{2}\right]+\lambda_{T_{r}G_{\beta }}\left[G_{\beta }\right]\right)\left[T_{N}\right]-\left(\delta_{T_{r}\mu_{1}}\left[\mu_{1}\right]+\delta_{T_{r}}\right)\left[T_{r}\right] \end{array}$$

(5) $$\begin{array}{cc} \frac{d\left[D_{N}\right]}{dt}= & A_{D_{N}}-\left(\lambda_{DH}\left[H\right]+\lambda_{DC}\left[C\right]\right)\left[D_{N}\right]-\left(\delta_{DH}\left[H\right]+\delta_{D}\right)\left[D_{N}\right] \end{array}$$

(6) $$\begin{array}{cc} \frac{d\left[D\right]}{dt}= & \left(\lambda_{DH}\left[H\right]+\lambda_{DC}\left[C\right]\right)\left[D_{N}\right]-\left(\delta_{DH}\left[H\right]+\delta_{DC}\left[C\right]+\delta_{D}\right)\left[D\right] \end{array}$$

(7) $$\begin{array}{cc} \frac{d\left[M\right]}{dt}= & \left(\lambda_{M\mu_{2}}\left[\mu_{2}\right]+\lambda_{MI_{\gamma }}\left[I_{\gamma }\right]+\lambda_{MT_{h}}\left[T_{h}\right]\right)\left(M_{0}-\left[M\right]\right)-\delta_{M}\left[M\right] \end{array}$$

(8) $$\begin{array}{cc} \frac{d\left[C\right]}{dt}= & \left(\lambda_{C}+\lambda_{C\mu_{1}}\left[\mu_{1}\right]\right)\left[C\right]\left(1-\frac{\left[C\right]}{C_{0}}\right)-\left(\delta_{CG_{\beta }}\left[G_{\beta }\right]+\delta_{CI_{\gamma }}\left[I_{\gamma }\right]+\delta_{CT_{C}}\left[T_{C}\right]+\delta_{C}\right)\left[C\right] \end{array}$$

(9) $$\begin{array}{cc} \frac{d\left[N\right]}{dt}= & \alpha_{NC}\left(\delta_{CG_{\beta }}\left[G_{\beta }\right]+\delta_{CI_{\gamma }}\left[I_{\gamma }\right]+\delta_{CT_{C}}\left[T_{C}\right]+\delta_{C}\right)\left[C\right]-\delta_{N}\left[N\right] \end{array}$$

(10) $$\begin{array}{cc} \frac{d\left[H\right]}{dt}= & \lambda_{HN}\left[N\right]+\lambda_{HM}\left[M\right]+\lambda_{HT_{h}}\left[T_{h}\right]+\lambda_{HT_{C}}\left[T_{C}\right]+\lambda_{HT_{r}}\left[T_{r}\right]-\delta_{H}\left[H\right] \end{array}$$

(11) $$\begin{array}{cc} \frac{d\left[\mu_{1}\right]}{dt}= & \lambda_{\mu_{1}T_{h}}\left[T_{h}\right]+\lambda_{\mu_{1}M}\left[M\right]+\lambda_{\mu_{1}D}\left[D\right]-\delta_{\mu_{1}}\left[\mu_{1}\right] \end{array}$$

(12) $$\begin{array}{cc} \frac{d\left[\mu_{2}\right]}{dt}= & \lambda_{\mu_{2}M}\left[M\right]+\lambda_{\mu_{2}D}\left[D\right]+\lambda_{\mu_{2}T_{r}}\left[T_{r}\right]-\delta_{\mu_{2}}\left[\mu_{2}\right] \end{array}$$

(13) $$\begin{array}{cc} \frac{d\left[I_{\gamma }\right]}{dt}= & \lambda_{I_{\gamma }T_{h}}\left[T_{h}\right]+\lambda_{I_{\gamma }T_{C}}\left[T_{C}\right]+\lambda_{I_{\gamma }M}\left[M\right]-\delta_{I_{\gamma }}\left[I_{\gamma }\right] \end{array}$$

(14) $$\begin{array}{cc} \frac{d\left[G_{\beta }\right]}{dt}= & \lambda_{G_{\beta }M}\left[M\right]+\lambda_{G_{\beta }T_{r}}\left[T_{r}\right]-\delta_{G_{\beta }}\left[G_{\beta }\right] \end{array}$$

As it has been mentioned in [48], this system includes 14 variables and 59 parameters, where λ parameters correspond to proliferation, activation and production rates, while δ parameters denote the degradation and cell death rates. The parameters $A_{T_{N}}$ and $A_{D_{N}}$ respectively are the production rates of naive T-cells and dendritic cells (D), and $M_{0}$ and $C_{0}$ are the total density of macrophages (naive and activated together) and cancer cells’ maximum capacity, respectively.

To this given system, we add the individual interactions between the three drugs of the FOLFIRI regimen - Leucovorin (Folinic Acid), Fluorouracil (5-FU), and Irinotecan; and the variables of the above system. The metabolism and action pathways of these three drugs are complex, involving a number of different molecules and enzymes. Therefore, even though we initially attempted to have a comprehensive system with all of their pharmacodynamical reactions, due to the lack of available parameter values in research, we decided to simplify the model. Our condensed system focuses on the overall change in the drug concentrations and their effect on cells in the tumor microenvironment. The final condensed interaction network between FOLFIRI and the colon cancer environment is given in Figure 1.

Since we are adding the drugs to the system already developed in [48], we employ the same formula as given by the mass action law for defining the terms in our ordinary differential equations. Namely, for any biochemical process A+B→C, the differential equation for *C* is given by $\frac{dC}{dt}=\lambda AB$, where λ is the production rate of *C* [48,92,93]. Similarly, an inhibition process D⊣E is given by $\frac{dE}{dt}=-\delta DE$, where δ is the inhibition rate of *E*. Additionally, alphas (α) are included as constant parameters in the drug equations to necessarily differentiate between the inhibition rates on the targeted cells and the decay effect on the drug concentration itself. In the following, we explain the derived equations for the dynamics of each drug and the cells effected by them, with concentrations given in time, per unit day (changes to the equations from [48] have been highlighted in bold).

#### 2.1.1. Cancer & Necrotic Cells

We model the effects of FOLFIRI drugs (5-FU, Leucovorin, and Irinotecan) on cancer by modifing the Equation (8) in the following way.

(15) $$\begin{array}{cc} \frac{d[C]}{dt}= & \left(\lambda_{C}+\lambda_{C\mu_{1}}\left[\mu_{1}\right]\right)\left[C\right]\left(1-\frac{\left[C\right]}{C_{0}}\right)-(\delta_{CG_{\beta }}\left[G_{\beta }\right]+\delta_{CI_{\gamma }}\left[I_{\gamma }\right]+\delta_{CT_{C}}\left[T_{C}\right]+\delta_{C}\\ \; & +\delta_{C5\mathrm{fu}}\left[5\mathrm{FU}\right]+\delta_{C5\mathrm{fu}I_{\gamma }}\left[5\mathrm{FU}\right]\left[I_{\gamma }\right]-\delta_{5\mathrm{fu}M}\left[5\mathrm{FU}\right]\left[M\right]\\ \; & +\delta_{C\mathrm{LV}5\mathrm{fu}}\left[5\mathrm{FU}\right]\left[\mathrm{LV}\right]+\delta_{C\mathrm{Ir}}\left[\mathrm{Ir}\right])\left[C\right], \end{array}$$

where the decay rates represent the following: $\delta_{C5fu}$ and $\delta_{CIr}$ are the direct cytotoxic effects of 5-FU and Irinotecan respectively; $\delta_{C5fuI_{\gamma }}$ is the rate of cancer cell death due to the increased activation of 5-FU by IFN-γ; and lastly $\delta_{CLV5fu}$ is the combined inhibitory effect of 5-FU and Leucovorin.

Note, 5-FU causes damage to cancer cells by inhibiting essential processes in DNA and RNA synthesis [28,29]. This is done by two main pathways, either by the missincorporation of fluoronucleotides in both RNA and DNA, or by inhibiting the enzyme thymidylate synthase (TYMS), which is a crucial component of DNA replication and repair [28,29,94]. These pathways are aided by IFN-γ, which up-regulates the activities of 5-FU anabolic enzymes, increasing its cytotoxic effect [29]. Hence, Equation (15) includes the terms $\delta_{C5\mathrm{fu}}\left[5\mathrm{FU}\right]\left[C\right],\;\delta_{C5\mathrm{fu}I_{\gamma }}\left[5\mathrm{FU}\right]\left[I_{\gamma }\right]\left[C\right]$. Meanwhile, macrophages have been shown to decrease the inhibitory effect of 5-FU on cancer cells [95,96,97]; modeled by the term $\delta_{5\mathrm{fu}M}\left[5\mathrm{FU}\right]\left[M\right]\left[C\right]$ in the above Equation (15).

Leucovorin is often administered simultaneously with 5-FU in the treatment of colon cancer, because it has been shown to increase the efficacy of 5-FU and the overall survival rate in patients [94,98,99]. This effect is due to the fact that it helps to further stabilize the complex formed between TYMS and the 5-FU derivative, and thus increasing the retention of 5-FU toxicity [29,100]. Therefore, the effect of Leucovorin on cancer is modeled by the term $\delta_{C\mathrm{LV}5\mathrm{fu}}\left[5\mathrm{FU}\right]\left[\mathrm{LV}\right]\left[C\right]$ in Equation (15).

The effect of Irinotecan on cancer is modeled by $\delta_{C\mathrm{Ir}}\left[\mathrm{Ir}\right]\left[C\right]$, because Irinotecan prevents DNA replication by inhibiting the topoisomerase 1 gene (TOP1) causing subsequent cell death [101,102,103]. Although used as a part of the FOLFIRI regimen, it is also given individually in the treatment of cancer.

Consequently, the equation for necrotic cells becomes:

(16) $$\begin{array}{cc} \frac{d[N]}{dt}= & \alpha_{NC}{(\delta }_{CG_{\beta }}\left[G_{\beta }\right]+\delta_{CI_{\gamma }}\left[I_{\gamma }\right]+\delta_{CT_{C}}\left[T_{C}\right]+\delta_{C}\\ & +\delta_{C5\mathrm{fu}}\left[5\mathrm{FU}\right]+\delta_{C5\mathrm{fu}I_{\gamma }}\left[5\mathrm{FU}\right]\left[I_{\gamma }\right]-\delta_{5\mathrm{fu}M}\left[5\mathrm{FU}\right]\left[M\right]\\ + & \delta_{C\mathrm{LV}5\mathrm{fu}}\left[5\mathrm{FU}\right]\left[\mathrm{LV}\right]+\delta_{C\mathrm{Ir}}\left[\mathrm{Ir}\right])\left[C\right]-\delta_{N}\left[N\right] \end{array}$$

#### 2.1.2. T-Cells

Within the tumor-microenvironment, 5-FU is known to help in the activation of T-cells, along with dendritic cells [104], and Irinotecan depletes the number of T-reg cells [105]. As reported in [104], the interaction between 5-FU, dendritic cells and T-cells is rather complex, but there is an overall increase in the activation and generation of helper T-cells and cytotoxic cells due to the indirect transfection of dendritic cells by 5-FU, modelled by introducing activation rates $\lambda_{T_{h}D5fu}$ and $\lambda_{T_{C}D5fu}$ into the Equations (2) and (3), respectively.

(17) $$\begin{array}{cc} \frac{d[T_{h}]}{dt}= & \left(\lambda_{T_{h}D}\left[D\right]+\lambda_{T_{h}D5\mathrm{fu}}\left[5\mathrm{FU}\right]\left[D\right]+\lambda_{T_{h}M}\left[M\right]+\lambda_{T_{h}\mu_{1}}\left[\mu_{1}\right]\right)\left[T_{N}\right]\\ - & \left(\delta_{T_{h}\mu_{2}}\left[\mu_{2}\right]+\delta_{T_{h}T_{r}}\left[T_{r}\right]+\delta_{T_{h}}\right)\left[T_{h}\right] \end{array}$$

(18) $$\begin{array}{cc} \frac{d[T_{C}]}{dt}= & \left(\lambda_{T_{C}T_{h}}\left[T_{h}\right]+\lambda_{T_{C}D}\left[D\right]+\lambda_{T_{C}D5\mathrm{fu}}\left[5\mathrm{FU}\right]\left[D\right]\right)\left[T_{N}\right]\\ - & \left(\delta_{T_{C}\mu_{2}}\left[\mu_{2}\right]+\delta_{T_{C}T_{r}}\left[T_{r}\right]+\delta_{T_{C}}\right)\left[T_{C}\right] \end{array}$$

In [48], activation rates for T-cells were made proportional to the density of naive T-cells in order to help stabilize the system. Therefore, we also add the above activation rates to the Equation (1).

(19) $$\begin{array}{cc} \frac{d[T_{N}]}{dt}= & A_{T_{N}}-\left(\lambda_{T_{h}D}\left[D\right]+\lambda_{T_{h}D5\mathrm{fu}}\left[5\mathrm{FU}\right]\left[D\right]+\lambda_{T_{h}M}\left[M\right]+\lambda_{T_{h}\mu_{1}}\left[\mu_{1}\right]\right)\left[T_{N}\right]\\ & -\left(\lambda_{T_{C}T_{h}}\left[T_{h}\right]+\lambda_{T_{C}D}\left[D\right]+\lambda_{T_{C}D5\mathrm{fu}}\left[5\mathrm{FU}\right]\left[D\right]\right)\left[T_{N}\right]\\ & -\left(\lambda_{T_{r}T_{h}}\left[T_{h}\right]+\lambda_{T_{r}\mu_{2}}\left[\mu_{2}\right]+\lambda_{T_{r}G_{\beta }}\left[G_{\beta }\right]\right)\left[T_{N}\right]-\delta_{T_{N}}\left[T_{N}\right] \end{array}$$

Among the major effects of Irinotecan on the tumor microenvironment, it is also shown to be the reduction of the abundance of regulatory T-cells [105]. There are other sources that report a significant reduction in T-regs after the chemotherapy using FOLFIRI [106].

(20) $$\frac{d[T_{r}]}{dt}=\left(\lambda_{T_{r}T_{h}}\left[T_{h}\right]+\lambda_{T_{r}\mu_{2}}\left[\mu_{2}\right]+\lambda_{T_{r}G_{\beta }}\left[G_{\beta }\right]\right)\left[T_{N}\right]-\left(\delta_{T_{r}\mu_{1}}\left[\mu_{1}\right]+\delta_{T_{r}}+\delta_{T_{r}\mathrm{Ir}}\left[\mathrm{Ir}\right]\right)\left[T_{r}\right]$$

#### 2.1.3. 5-FU & Leucovorin

Thus, combining the above individual interactions with 5-FU, the overall effect on the 5-FU concentration can be modeled by

(21) $$\begin{array}{cc} \frac{d[5FU]}{dt}= & A_{inj}^{5fu}\left(t\right)-\alpha_{5fu}{(\delta }_{C5fu}\left[5FU\right]+\delta_{C5fuI_{\gamma }}\left[5FU\right]\left[I_{\gamma }\right]-\delta_{5fuM}\left[5FU\right]\left[M\right]\\ \; & +\delta_{CLV5fu}\left[5FU\right]\left[LV\right])\left[C\right]-\delta_{5fuD}\left[5FU\right]\left[D\right]-\delta_{5fu}\left[5FU\right] \end{array}$$

Along with the parameters from the equation for cancer cells, 5-FU is modelled with additional parameters, $\delta_{5fuD}$ for the amount of 5-FU used to help dendritic cells activate T helper cells, $\delta_{5fu}$ to represent the elimination rate (about 80% of 5-FU is consumed in the liver [28,107,108]), $\alpha_{5fu}$ as the amount of 5-FU used to kill a unit of cancer cells and $A_{inj}^{5fu}\left(t\right)$ is a function in time to model the repetitive cycle of 5FU dosages.

(22) $$\frac{d[LV]}{dt}=A_{inj}^{LV}\left(t\right)-\delta_{LV}\left[LV\right]-\alpha_{LV}\;\delta_{CLV5fu}\left[C\right]\left[5FU\right]\left[LV\right]$$

Leucovorin is similarly modelled with $A_{inj}^{LV}\left(t\right)$, a function for the dosage intake, a natural decay rate represented by $\delta_{LV}$, $\delta_{CLV5fu}$ from the cancer equation since Leucovorin increases the cytotoxic effect of 5-FU and $\alpha_{LV}$ the effectiveness of Leucovorin in killing cancer cells.

#### 2.1.4. Irinotecan

Dynamics of Irinotecan is modeled in a similar way with a natural decay rate of Irinotecan denoted by $\delta_{Ir}$, the death rate of cancer cells by Irinotecan as $\delta_{CIr}$ and the effectiveness of killing cancer cells by the constant $\alpha_{CIr}$. Since we also know that Irinotecan depletes T-reg cells [105], we include the parameters $\delta_{TrIr}$, the depletion of T-reg cells by Irinotecan, and $\alpha_{IrTr}$, the effectiveness of Irinotecan in the depletion of T-reg cells.

(23) $$\frac{d[Ir]}{dt}=A_{inj}^{Ir}\left(t\right)-\alpha_{IrC}\;\delta_{CIr}\left[C\right]\left[Ir\right]-\alpha_{IrT_{r}}\;\delta_{T_{r}Ir}\left[Ir\right]\left[T_{r}\right]-\delta_{Ir}\left[Ir\right]$$

### 2.2. Non-Dimensionalization

Non-dimensionalization is used for additional numerical stability and to eliminate scale dependence [48]. The original system in [48] was non-dimensionalized by considering a non-dimensional variable $\bar{X}$ such that,

$$\bar{X}=\frac{X}{X^{\infty }}$$

for each variable *X*, where $X^{\infty }$ is its steady-state value. For the new variables, namely the FOLFIRI drugs, we introduce new non-dimensional variables in the form of $\bar{D}$ for each drug *D*, defined as:

$$\bar{D}=\frac{\delta_{D}\left[D\right]}{{\langle A_{inj}^{D}\rangle }_{daily\;median}},$$

where $\delta_{D}$ is its natural decay rate and ${\langle A_{inj}^{D}\rangle }_{daily\;median}$ is its daily median dose per cycle from patient data (See Appendix A for further details).

### 2.3. Data of the Model

There are several popular tumor deconvolution methods to estimate immune cell frequencies using the gene expression profile of the tumors, and it has been shown in recent studies that CIBERSORTx method [109] has the highest accuracy among these methods [110,111,112]. In this study, we download RSEM normalized RNA-seq gene expression profiles in $\log_{2}$ scale of the primary tumors of the 329 colon cancer patients from the TCGA project of COAD from UCSC Xena web portal [113] and transform it to the linear space. Then, we apply CIBERSORTx B-mode on the gene expression data to estimate immune cells frequencies.

**Figure 2:**
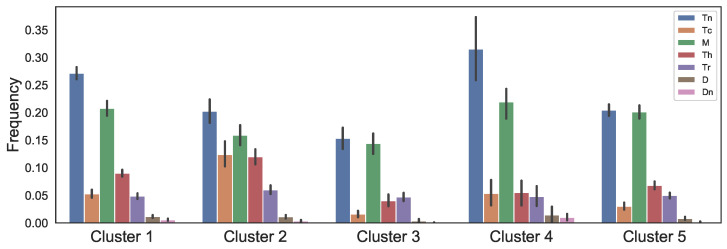
Model immune cells fractions. Clusters are obtain based on variations in 22 immune cell types of colonic tumor.

In our previous study, K-means clustering of colon tumors based on their immune cells’ frequencies indicates that there are five distinct immune patterns of colonic tumors [48]. In this paper, we investigate the effect of FOLFIRI drugs on the same five distinct clusters. In each cluster, average immune cell frequencies that we use in the dynamical model have been shown in Figure 2, where the vertical bars show the standard deviations.

The data used in Figure 2 only gives us the ratio of immune cells so that we download TCGA biospecimen data from GDC portal that includes tumor dimension and necrotic cell percentage of the tumors to obtain the values of the model variables as described below.

Assuming the average amount of cancer cells is twice the average amount of total immune cells (TIC) and using the given necrosis percentage from TCGA biospecimen data, the average ratio of immune cells: cancer cells: necrotic cells is approximately 0.3:0.6:0.1 for colon cancer tumors. Also, we define size of tumor for each patient (*P*) as the product of the longest and the shortest dimension of the tumor, and total cell density (TCD) is assumed to be proportional to the size of the tumor.

$${\mathrm{TCD}}_{P}=\alpha_{dim}\frac{\mathrm{tumor}\;\mathrm{size}(P)}{\frac{1}{K}\sum_{\mathrm{all}\;P}\mathrm{tumor}\;\mathrm{size}\left(P\right)}$$

Using TCD and necrotic cell percentage ($N_{p}$), we calculate the value of N and C in the following way:

$$N=\mathrm{TCD}.N_{p}\;\;,\;\;C=\frac{2}{3}\mathrm{TCD}\left(1-N_{p}\right)\;\;\mathrm{and}\;\;\mathrm{TIC}=0.5C.$$

For scaling factor $\alpha_{dim}$, we choose 7.5 × 10${}^{4}$ to approximately match the average density of cancer cells for all patients to be 4.5 × 10${}^{4}$ cells/cm${}^{3}$, which is reported in [114]. We determine the smallest tumors in each cluster and use their values as the initial conditions of the model for each cluster. As we solve the non-dimensionalized system, Table 2 shows the initial values of the non-dimensionalized variables, i.e., $X/X^{\infty }$ for each cluster.

#### Treatment Data

For modeling FOLFIRI, we have downloaded the clinical drug data from the GDC portal that includes prescribed treatment information such as drug dosages, number of cycles of a specific treatment the patient received, days to start treatment and days to end treatment. To model each cluster, we have used patients average prescribed treatment information (Table 3). Note that minimum dosages values are used in parameter estimation of the model explained with more details in Appendix A.

### 2.4. Numerical Methods

To solve the system and study the dynamics of the variables, the previously developed code in [48] is modified to include the new equations, variables and parameters. The values for the parameters of the ODE system are either derived from research or by making appropriate assumptions based on biological information. The code uses SciPy solve_ivp function in python [115] to solve the system and the drug information is obtained from the treatment data as given in Table 3, to be used as the initial infusion rates. The infusion step function is modelled after the FOLFIRI chemotherapy regimen given in [116]. FOLFIRI is administered first with one hour drip of Irinotecan followed by one hour drip of Leucovorin, after which 5-FU is continuously infused into the bloodstream for 46 h. This treatment is repeated for the designated ‘number of cycles’, each cycle lasting over the period of the ‘cycle length’.

### 2.5. Sensitivity Analysis

To analyze the effect of parameter values on the dynamics of the system, we perform sensitivity analysis [117,118,119]. For the system $\frac{dX}{dt}=F\left(X,\;\theta ,\;t\right)$ consider (first order) sensitivity *S* of non-dimensional solution *X* with respect to the model parameters $\theta ={\left\{\theta_{i}\right\}}_{i=\bar{1,\;N}}$ to be defined as a vector

$$S_{i}=\frac{dX}{d\theta_{i}},\;i=\bar{1,\;N}.$$

As within the introduced model, the effects of the treatment do not extend to the steady state. Therefore, we consider time-dependent sensitivity satisfying the equation

$$\frac{dS_{i}}{dt}=\frac{\partial F}{\partial \theta_{i}}+\frac{\partial F}{\partial X}S_{i}.$$

Additionally, we look at the “relative” sensitivity given by the formula

$${\bar{S}}_{i}\left(t\right)=S_{i}\left(t\right)\frac{\theta_{i}}{X(t)}.$$

Relative sensitivity approach is commonly used in metabolic control analysis for biological reaction networks [120]. Then, for finite time *T*, we consider average sensitivity of each type:

$$S_{i}=\frac{1}{T}\int_{0}^{T}S_{i}\left(t\right)dt,\;{\bar{S}}_{i}=\frac{1}{T}\int_{0}^{T}{\bar{S}}_{i}\left(t\right)dt.$$

## 3. Results

### 3.1. Dynamics

**Figure 3:**
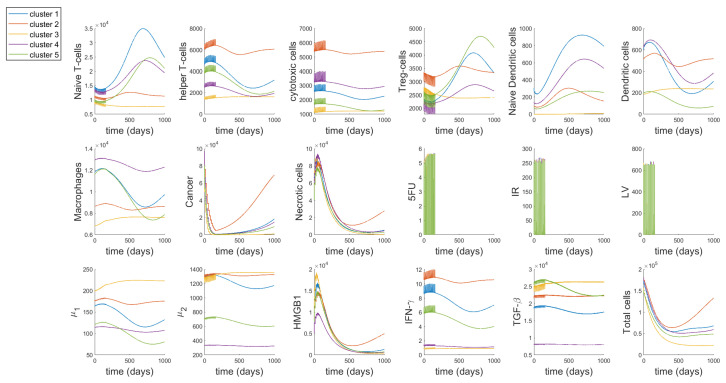
Whole system dynamics with initial conditions as median drug doses from patient data and steady-state values for the cells. The different colour lines depict the different dynamics of each cluster. ‘Total cells’ is the sum of all immune, cancer and necrotic cells excluding cytokines.

We study the individual dynamics of each of the 17 variables and ‘Total cells’ (Immune cells, cancer and necrotic cells), with most of the parameter values obtained from [48] (using steady-state assumptions) and the new 19 parameters derived from parameter assumptions (as given in Appendix A). The drug inputs are the median drug dosages from patients treated with FOLFIRI—770, 725 and 300 mg of 5-FU, Leucovorin and Irinotecan, respectively. The average number of cycles and cycle length respectively are 12 and 14, as given in Table 3. In Figure 3, the initial conditions for the cells in each cluster are taken to be their steady-state values from [48], which correspond to the values of large tumors in each cluster.

Here, we study two different periods in the dynamics, during treatment and about two years after treatment has been stopped. We can observe that during the treatment, there is a decline in the number of Naive T-cells, T-reg cells, TGF-β and cancer cells. On the other hand, helper T-cells, cytotoxic cells, necrotic cells, HMGB1 and IFN-γ increase during treatment. Macrophages and $\mu_{1}$ group of cytokines increase only by a small amount during treatment while $\mu_{2}$ group of cytokines decrease slightly and this effect is most prominent for cluster 3 as compared to the other clusters. The effect of the treatment on both naive and active dendritic cells is not distinctly discernible, although we are able to observe smaller spikes during the time of treatment.

Based on our ODE system, we can infer that the increase in helper T-cells and cytotoxic cells is due to the activation rates $\lambda_{T_{h}5fuD}$ and $\lambda_{T_{C}5fuD}$, that are modelled after the indirect activation of T-cells by dendritic cells and 5-FU [104]. On the other hand, the decline in T-reg cells must be due to the inhibitory effect of Irinotecan on T-reg cells, modelled in Equation (20) by the parameter $\delta_{T_{r}Ir}$. We have described macrophages to have an inhibitory effect on the cytotoxic action of 5-FU, but they themselves are not affected by this interaction, because this is the only interaction modelled between macrophages and the drugs in our network (as seen in Figure 1). Note, the small spikes of macrophages may be attributed to the activation rate of macrophages by T-helper cells, $\lambda_{MT_{h}}$, as 5-FU has an interaction with T-helper cells. Since we include the activation rates for helper and cytotoxic T-cells in the equation for naive T-cells (Equation (19)), the decrease in the density of naive cells is also reasonable. The small effect on dendritic cells, both naive and active can be explained by the parameter $\lambda_{DC}$, since this parameter is linked to the density of cancer cells. For all the clusters, there is a rapid decline in the cancer cell density during treatment. Cluster 3 reaches the minimum cancer value among the clusters which is quite close to zero. Since the amount of necrotic cells increases with the decrease in cancer cells, it is reasonable to see it increases during treatment. Moreover, IFN-γ is not directly affected by any of the drugs, however, since helper T-cells increase during treatment this must also increase the value of IFN-γ. Cytokines, $\mu_{1}$, $\mu_{2}$, HMGB1 and TGF-β are all indirectly affected as per their interactions with macrophages, T-cells and dendritic cells in the tumor network Figure 1.

**Figure 4:**
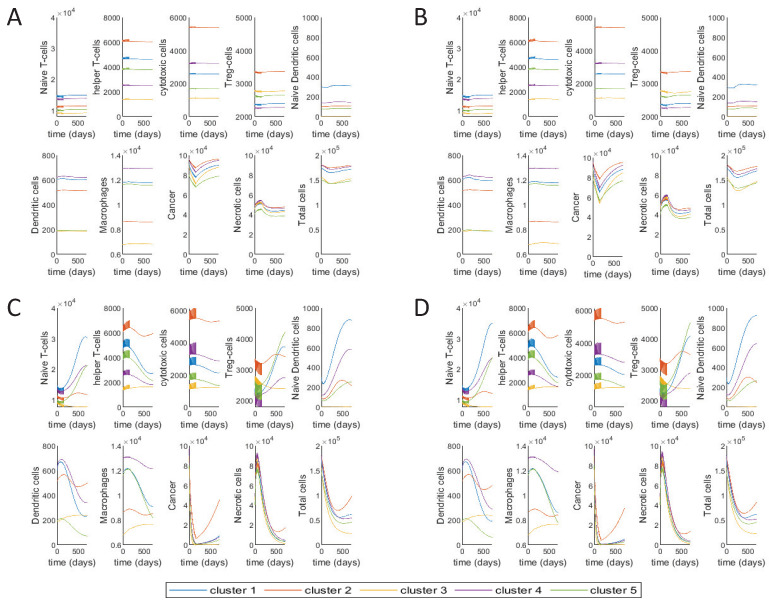
Varying combinations of the FOLFIRI drugs. In (**A**), we use the median dosage for 5-FU as the initial infusion dose, keeping the values of Leucovorin and Irinotecan zero. In (**B**), we add the median dose of Leucovorin to that of 5-FU, keeping Irinotecan dose at zero. Similarly in (**C**), 5-FU and Irinotecan are infused at the median dosage, while keeping Leucovorin at zero. (**D**) is the model results obtained from infusing all three drugs at median dosages. Median dosages are given in Table 3.

After the treatment stops, all the cells continue growing as per the model without any treatment, which is basically the system in [48], eventually reaching steady state. While the clusters generally show a common pattern, we are able to observe some unique behaviour in certain cell dynamics. Cluster 3 shows a slight increase in helper T-cells, cytotoxic cells, dendritic cells, macrophages, $\mu_{1}$, $\mu_{2}$ and TGF-β, right after treatment and remains almost flat in its growth until the end of time period, decreasing again by a small amount. These combined changes could explain the decline in naive T-cells, as can be observed through Equation (19), and subsequently the decline in T-reg cells modeled by Equation (20), which depends on naive T-cells. Cluster 3 also reaches the lowest density of cancer cells, necrotic cells and ‘Total cells’ among all the clusters at the lowest point as well as by the end of the time period. While cluster 2 has the highest number of cancer cells and ‘Total cells’ at the lowest point and by the end of the time period. Importantly, comparing our results to that in [48], we can observe that cluster 3 shows a unique behavior for the model without treatment. In Figures 4 and 5 in [48], cluster 3 also shows similar patterns to that observed in our model with treatments, which further confirms that while the cell dynamics change during treatment, it does not change the overall dynamics.

### 3.2. Different Treatment Options

We investigate the individual and combined effect of the three drugs by plotting the cell dynamics with different combinations of the drugs. The initial conditions for the cells in each cluster are taken to be the steady state values, i.e., the values of large tumors [48]. For Figure 4A, we study the individual effect of 5-FU by keeping the other two drug values at zero. For Figure 4B, we study the combined effect of 5-FU and Leucovorin by keeping the Irinotecan dose at zero. For Figure 4C, we now study the combination of 5-FU and Irinotecan, keeping Leucovorin dose at zero. Finally, in Figure 4D, we study the combined effect of the three FOLFIRI drugs together. The drug values are their corresponding median dose values as given in Table 3. The number of cycles and cycle length respectively are 12 and 14 for all the drug combinations (given in Table 3).

There is not much differences in the dynamics of key players when using 5-FU alone versus 5-FU plus Leucovorin, except for a clear decrease only during and right after the treatments in cancer cells and consequently ‘Total cells’, which is to be expected since Leucovorin aids 5-FU in killing cancer cells but does not have any other direct interaction with other cells in the tumor microenvironment, as can be seen in Figure 1. With the addition of Irinotecan to 5-FU, there is a dramatic change in most of the cells, and especially T-reg cells. Adding Irinotecan changes the dynamics of T-reg and dendritic cells leading to slower tumor recurrence, especially for tumors in cluster 3. This is also an expected result, since Irinotecan is assumed to be 40% efficient in killing cancer cells, which is much higher than the other two drugs. Irinotecan also specifically targets T-reg cells, as is modelled by the parameter $\delta_{TrIr}$ in the Equation (20).

### 3.3. Varying Treatment Start Time

We also investigate the effect of the treatment start time on dynamics of immune and cancer cells, since different patients start chemotherapy at different stages of their cancer. The initial conditions for the cells and cytokines for each cluster is obtained from taking the treatment data for the patient with the smallest tumor in each cluster (Table 2). The initial conditions for the drugs are taken to be their median dosages and the average for the number of cycles and cycle length as given in Table 3.

**Figure 5:**
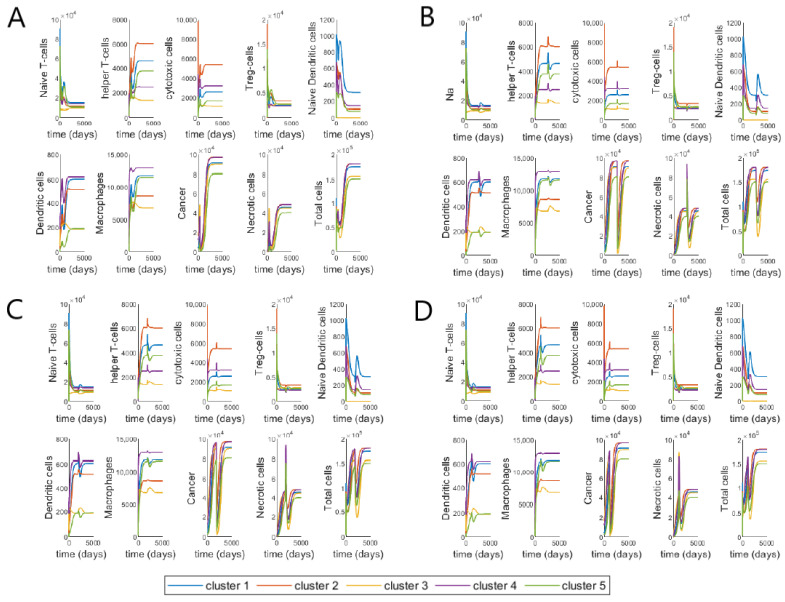
Varying treatment start-time. In the corresponding (**A**–**D**), the treatment is given after 1, 3, 5 and 7 years respectively. Seven years is the approximate time for the cancer to reach the steady state.

Giving the treatment at a later time, delays the time it takes for the cells to reach their steady states. As discussed previously, the cell dynamics are effected only during treatment, but the treatment does not change the overall dynamics, and we can observe the same effect here. In Figure 5A, cluster 5 has the lowest number of cancer cells after treatment, while in the other Figure 5B–D when the treatment is given after 3 years or later, cluster 3 reaches the lowest number of cancer cells after treatment. In Figure 5C,D, all cells seem to have already reached or very close to reaching their steady-states. Hence, there is not much of a difference between the dynamics in these cells between the plots C and D, except that the effect of the treatment is seen at a later time. In Figure 5D, since the treatment is given after about 7 years all the cells, including cancer and necrotic cells have already reached their steady states and therefore the dynamics is identical to that in Figure 3. As also observed in Figure 3 and Figure 4, cluster 3 shows some unique behavior compared to the other clusters. The number of dendritic cells in the other clusters is much higher than their initial value, but for cluster 3 it decreases after treatment and reaches to the same steady state value as cluster 5. Cluster 3 has the lowest number of total cells after the treatment in all plots, but cluster 5 has the lowest steady state value for cancer, necrotic and total cells.

### 3.4. Validating Model Using Patient Data

We have used clinical follow up data of the colon cancer patients downloaded from the GDC portal that includes tumor status and corresponding days of the follow up in different times to see if the number of cancer cells of the patients obtained from our model would match with their follow up data. Not every patients’ treatment information such as drug dosages and number of cycles are available, and there are only 4 patients who used Fluorouracil, Leucovorin and Irinotecan that we know their prescribed treatment information. For this reason, we validate our model with 36 patients that use Fluorouracil and Leucovorin. Note that most of these patients have also used other drugs such as Oxaliplatinum that are not included in the model.

We validate our model based on patients’ tumor status in their two different follow up days (Table 4). We consider patients who had only one follow up date in both groups. We exclude patients in the early follow up group, if their treatments have not been ended before their follow up day.

#### 3.4.1. Predicting Tumor Status—ROC Curve

**Figure 6:**
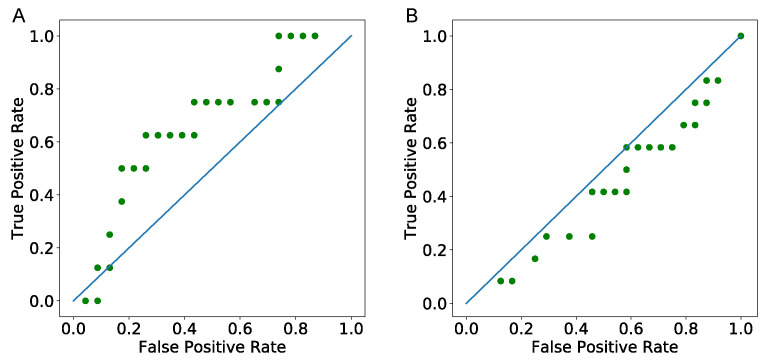
ROC Curves of the model. (**A**,**B**) are generated using predicted cancer cells number in the first day of follow up and the last day of follow up of the patients, and AUC values are 0.53 and 0.41, respectively.

Data includes the start date of treatments and the last follow up date that the patients’ tumor status has been recorded. Therefore, to validate the model, we use the predicted numbers of cancer cells from the model at the exact number of days after each patient’s start date of treatments that their tumor status has been recorded. We then define a range of threshold values using minimum and maximum predicted values to create a ROC curve and predict patients’ tumor status as tumor free if the estimated number of values are less than the threshold value. As seen in Figure 6, area under the curve (AUC) for the first follow up days ROC curve (Figure 6A) is greater than the one for the last follow up days ROC curve (Figure 6B). As this Figure shows, the model’s predictions are good for the first follow up dates, but not for the second follow up dates. The bad prediction of the model for the last follow up date might be due to the fact that these patients might have had some other treatments, including surgery between the first and the last time of follow-up.

#### 3.4.2. Individual Patients

We investigate the effect of dosages on the number of cancer and immune cells. From the same data set of 36 patients who were treated with 5-FU and Leucovorin, we choose one patient from each cluster. If the patient is ‘tumor-free’ on a particular follow-up day, then we expect that in our model results, the cancer on that day is at least less than the initial cancer value for small and medium size tumors and half of their initial values for large tumors. While for a ‘with-tumor’ patient, the number of cancer cells on the follow-up day should be greater than their initial values for small or medium size tumors or half of their initial values for large tumors. We find 10 patients satisfying these conditions among 15 patients from cluster 1, one patient among the 5 patients from each of the clusters 2 and 3 respectively, and 5 patients among the 10 patients from cluster 5. Note, there is no follow-up information available for cluster 4 and hence, cluster 4 patients have been excluded from the data. We investigate the effect of varying the dose on cancer and immune cells for some of patients that model predicts well to provide alternative optimal treatment options for these patients.

**Figure 7:**
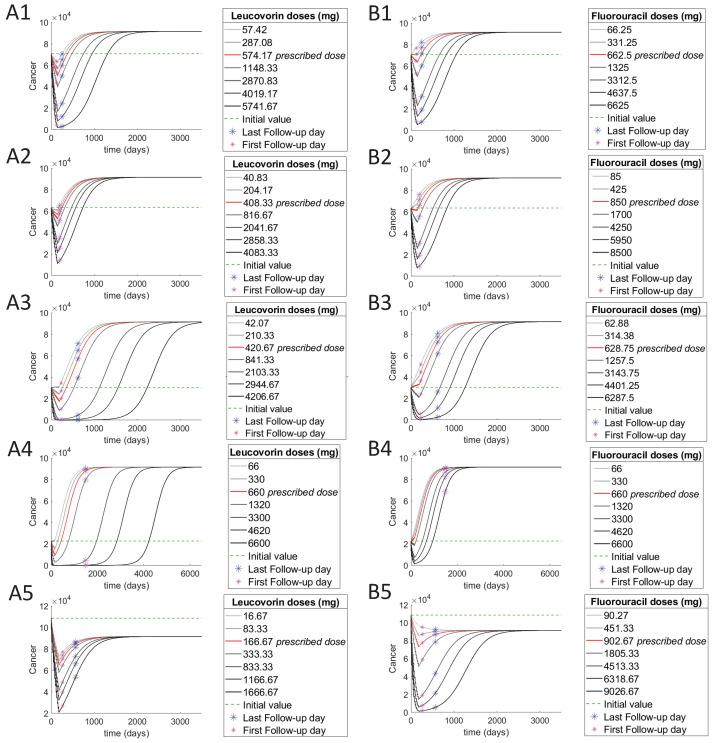
Cancer cells dynamics with varying doses for individual patients in each cluster. (**A**) are obtained by varying the Leucovorin dose while keeping the 5-FU dose constant. (**B**) are obtained by varying 5-FU and keeping the Leucovorin dose at zero. (**A1**,**B1**), (**A2**,**B2**), (**A3**,**B3**), and (**A5**,**B5**) respectively show the results of a tumor-free patient in the clusters 1, 2, 3 and 5. Note, the patient in cluster 3 (**A3**,**B3**) is tumor-free at the first follow-up day, but is with-tumor at the last follow-up day. (**A4**,**B4**) show the results of a with-tumor patient in the cluster 5.

The drug dosages and initial conditions for the cells used in the model are based on the individual patient’s treatment data. In Figure 7, sub-figures A are plotted by keeping the 5-FU dose constant at the patient’s administered dose, while Leucovorin is varied between a range of 0.1 and 10 times the prescribed dose. Sub-figures B are plotted by keeping the Leucovorin dose at zero while varying the 5-FU dose between a range of 0.1 and 10 times the patient’s prescribed dose.

Among the patients in cluster 1, we investigate the results for the patient ‘TCGA-CM-6172’, since we know from the treatment data that this patient was treated with only 5-FU and Leucovorin without the aid of any other drug. Therefore, this patient is an ideal candidate for validating our model. This patient was administered a 662.5 mg dose of 5-FU and 574.17 mg dose of Leucovorin in 12 cycles and is reported to be ‘tumor-free’ at both the first and last follow-up days. As can be observed in the Figure 7(A1), the cancer at both follow-up days is less than the initial cancer value and therefore the follow-up information matches our model results.

We investigate the effect of varying the dose for the patient ‘TCGA-A6-6141’ in cluster 2 whose follow-up data matches our model results. This patient is treated with a 850 mg dose of 5-FU and 408.3 mg of Leucovorin in 12 cycles. This patient has also been reported ‘tumor-free’ at the follow-up day, and this is consistent with our results shown in Figure 7(A2) obtained for cancer.

We plot the dynamics for the patient ‘TCGA-A6-6142’ in cluster 3 whose tumor status matches our model results. This patient was administered 628.75 mg of 5-FU and 420.67 mg of Leucovorin in 12 cycles. This patient is reported to be ‘tumor-free’ at the first follow-up day, but is reported to be ‘with-tumor’ at the last follow-up day. This can also be confirmed through Figure 7(A3), where for the prescribed dose, the cancer is lower than the initial value at the first follow up day but grows back again surpassing the initial value, and are much higher on the last follow-up day. The increase in the total immune cells with increasing doses is much higher for this patient. Figure 7(A3,B3) also indicate a significant delay in reaching steady state for cancer when the dose increases. From these sub-figures, we can also recommend a higher dose of Leucovorin at 2103.33 mg or 5-FU alone at 3143.76 mg, or higher to achieve ‘tumor-free’ results on the last follow-up day.

We investigate the dynamics of one ‘with-tumor’ patient from cluster 5. Patient ‘TCGA-G4-6303’ was administered a dose of 660 mg of 5-FU and Leucovorin in 6 cycles. Figure 7(A4) shows that at the prescribed dose, the cancer is higher than the initial value. Note, the patient’s prescribed dosage does not even reduce the number of cancer cells much during treatment. 5-FU alone, does not have any significant effect in reducing the cancer for this patient, we instead recommend a dose of 3300 mg of Leucovorin along with the original 5-FU dose, or higher to achieve ‘tumor-free’ results for a long period of time.

Although the cancer initially decreases with treatment, it grows back again after a period of time according to the tumor recurrence rate modelled into the system. And therefore as one can observe for the above patients, the steady state values are always higher than the initial value, which means that the patient may once again have cancer years after receiving treatment. However, depending on the age of the patient, the tumor might not reach a visible size in the patients’ life time. In general, the best outcome is to achieve a steady-state value for cancer that remains below the initial value. In order to demonstrate this, we plot the patient ‘TCGA-A6-6781’ from cluster 5, for whom, according our model, its steady state value is less than its initial value. This patient is treated with 902.67 mg of 5-FU and 166.67 mg of Leucovorin in 12 cycles. Although there is nothing distinctive to predict such results, in fact this patient has the highest initial cancer value among all the patients in the dataset. Interestingly, it seems 5-FU alone in a higher dose is a better treatment option for this patient, while for most patients the combination of 5-FU and Leucovorin works better than 5-FU alone.

#### 3.4.3. Effect of Sensitive Parameters

**Figure 8:**
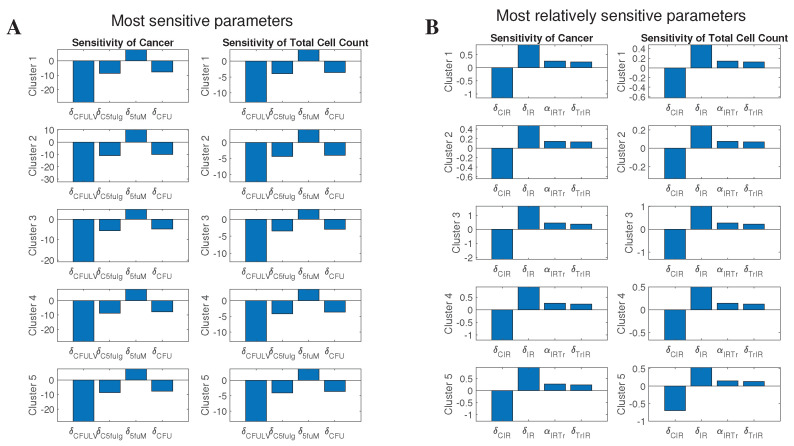
Sensitivity analysis. The first and second columns of (**A**) respectively present the results of non-dimensional sensitivity of cancer cell density and total cell density. (**B**) shows the relative sensitivity of the same quantities. Each row of plots shows the most sensitive parameters for each cluster of patients.

We perform sensitivity analysis of the non-dimensional system with respect to treatment-related parameters. Cells are assumed to be at their “no treatment” steady states (i.e., large tumors) for the initial conditions. The resulting time-averaged sensitivity for most sensitive parameters is presented on Figure 8.

These results show that the most sensitive parameter is $\delta_{C5fuLV}$, and $\delta_{5fuM}$ is the most sensitive among immune parameters. We investigate their effect on cancer cells and ‘Total cells’, by varying them between a range of 0.1 and 5 times their original value obtained from parameters’ assumptions, and plotting them against the number of cells at different time points. In these plots, the initial conditions for the cells were chosen to be their steady state values, i.e., values of large tumors.

**Figure 9:**
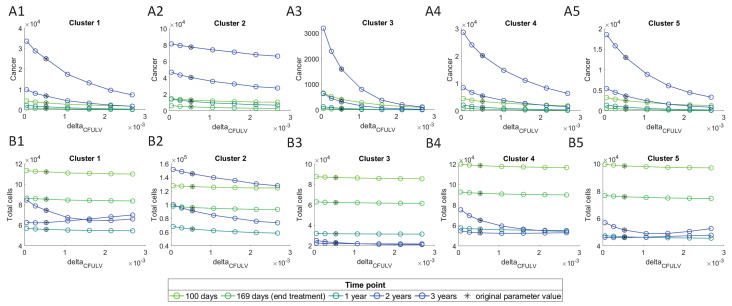
Cancer and total cells as a function of $\delta_{C5fuLV}$. (**A1**–**A5**) depict number of cancer cells and (**B1**–**B5**) depict number of total cells, at different time points.

Figure 9 shows a decline in the number of cancer cells with an increase in the $\delta_{C5fuLV}$ value, and this decline is observed to be smaller in cluster 2 as compared with the other clusters. The lowest number of cancer cells can be observed at 169 days, which is right after the treatment ends. Total cells seem to only be effected by increasing values at the 2nd and 3rd year time points, while for the other time-points there is barely any effect. This must be due to the fact that after treatment ends, the cancer starts to grow back again. In clusters 1 and 5, although the total number of cells first decreases at the year three, it increases at higher $\delta_{C5fuLV}$ values.

**Figure 10:**
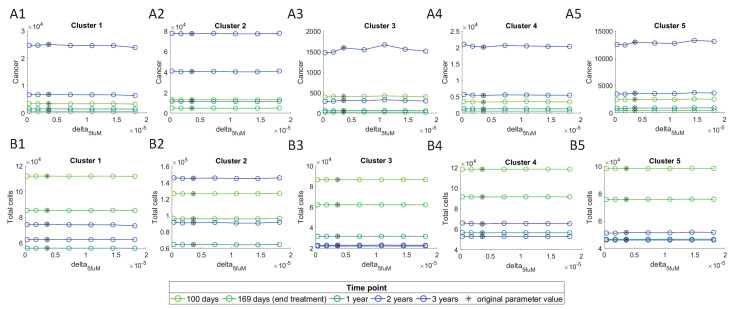
Cancer and total cells as a function of $\delta_{5fuM}$. (**A1**–**A5**) depict cancer cells and (**B1**–**B5**) depict Total cells, at different time points.

Varying $\delta_{5fuM}$ does not seem to have any significant change in the cancer or total cells (Figure 10), which is consistent with noticeably lower sensitivity level of this parameter compared to $\delta_{C5fuLV}$ (Figure 8). We similarly see the lowest number of cancer cells at 169 days, right after stopping the treatment, and the cancer increases after treatment.

## 4. Discussion

Cancer is a heterogeneous disease that includes different components such as immune cells, cancer cells or lymphatic vessels [121]. Drug resistance is one of the main problems in cancer studies [122]. Mechanisms or components of cancer are usually investigated one by one in traditional in vitro and in vivo studies. Although these studies provide valuable information about a mechanism, each of these studies alone is not able to provide necessary and sufficient information to explain cancer complexity [123]. Having more accessible biological experimental data set and new advances in tumor deconvolution methods lead to increase demand in data driven mathematical models that help us to model several mechanisms together and study the complexity of the system in a more effective way [124]. Tumor microenvironment components have an essential role in the explanation of poor prognosis and immune escape in CRC [21,22,23,125], and they have been used to explain chemotherapy and immunotherapy sensitivity in many studies [44,126,127]. In this study, we develop a data driven mathematical model that takes each tumor’s characteristic into consideration for the treatment. We have used interaction between TME components and drug mechanism to model the response to the FOLFIRI treatment in colon cancer using gene expression profiles of the CRC primary tumors to estimate immune patterns. Our results demonstrate how leucovorin increases the efficiency of 5-FU on cancer cells (Figure 4A,B) that has been shown in many studies [94,98,99]. Also, the impact of the combination of irinotecan, 5-FU, and leucovorin can be seen clearly on cancer cells in our model (Figure 4D) as reported in other studies [31,128]. In addition, we have applied treatment in different time points to show how cancer comes back aggressively if the treatment starts late (Figure 5).

The mathematical model demonstrates the relation between immune infiltration and drug’s effects on CRC primary tumors. Relative change in T-reg/T-helper ratio has been found as clinical index for response prediction; colon cancer patients with higher T-reg/Th ratio respond better to treatments [129]. Cluster 2 that has the lowest level of T-reg/T-helper ratio (Figure 2) is more resistant to FOLFIRI treatment since after the treatment the number of cancer cells increase faster and the values are not close to zero right after the treatment, while in other clusters the number of cancer cells approaches zero and their growth rate is slower (Figure 3). In contrast, the FOLFIRI treatment works better for cluster 3 that has the highest level of T-reg/T-helper ratio compared to the other clusters. Also, a decrease in regulatory T-cells after FOLFIRI treatment in colon cancer compared to pre-treatment level has been found to be associated with better survival months [130]. We observe a similar decrease for regulatory T-cells in tumors in cluster 3 that have a good response to the FOLFIRI treatment (Figure 3).

It has been observed that the number of T-reg cells significantly decreases for colon cancer patients who have a high number of regulatory T-cells before FOLFIRI treatment [106]. We divide patients into two groups based on their regulatory T-cells values as high T-reg and low T-reg. When we compare T-reg values before and after the treatment, we also see a decrease but it is not significant. In our validation data, there are only a few number of patients in the high T-reg group, and that might be the reason for not observing a significant p-value. It is important to mention that it would be ideal to use the gene expression of the patients after the treatment or data from patients who do not use other drugs rather than FOLFIRI to validate the model. However, follow up gene expression values are mostly unavailable, and we have only a small number of patients with all their treatment information available. Thus, we validate our model based on patients tumor status at follow up date. As we see in Figure 6, our model predicts much better for the first follow up data compare to the last follow up. That might be reasonable, because patients might have had other treatments such as surgery between their first and last time of follow ups.

In general, this work has some limitations that should be considered when these results are used. As mentioned above, patients included in the validation were also treated with other drugs such as oxaliplatin. Moreover, only 31 patients have early follow up data, and only 36 patients have late follow up data. Therefore, the validations have been done on a small number of patients who might have undergone other treatments. We have not had any data to validate the predictions of the model for the responses to Irinotecan.

Although our model has some limitation due to the lack of time course data, it presents valuable insight about the interactions between FOLFIRI treatment and the tumor micro-environment. Moreover, many studies can build upon on this one to provide the best treatment options for patients using only patients’ gene expression data. One way to improve this model is adding other chemotherapy options such as Oxaliplatinum, and different parameter fitting algorithm can be applied to increase the accuracy of the model [131,132,133,134]. Another possible way might be including other drug resistance factors in the model and extend it to a powerful model that considers other mechanisms and other types of patients’ data.

## Figures and Tables

**Table 1:** Model’s Variables. Names and descriptions of variables used in the model.

Variable	Name	Description
$T_{N}$	Naive T-cells	
$T_{h}$	Helper T-cells	
$T_{C}$	Cytotoxic cells	includes CD8+ T-cells and NK cells
$T_{r}$	Regulatory T-cells	
$D_{n}$	Naive dendritic cells	
*D*	Activated dendritic cells	antigen presenting cells
*M*	Macrophages	
*C*	Cancer cells	
*N*	Nectrotic cells	
*H*	HMGB1	
$\mu_{1}$	Carcinogenic cytokines	includes effects of IL-6, IL-17, IL-21 and IL-22
$\mu_{2}$	Immunosuppresive agents	includes effects of IL-10 and CCL20
$I_{\gamma }$	IFN-γ	
$G_{\beta }$	TGF-β	
5-FU	Fluorouracil	
Ir	Irinotecan	
LV	Leucovorin	

**Table 2:** The smallest tumor initial conditions in each cluster. Values of initial conditions for the dimensionless system are derived from the patients with the smallest tumor size.

Cluster	$T_{N}/T_{N}^{\infty }$	$T_{h}/T_{h}^{\infty }$	$T_{C}/T_{C}^{\infty }$	$T_{r}/T_{r}^{\infty }$	$D_{N}/D_{N}^{\infty }$	$D/D^{\infty }$	$M/M^{\infty }$
1	3.66 × 10${}^{-2}$	4.90 × 10${}^{-2}$	9.67 × 10${}^{-2}$	2.70 × 10${}^{-2}$	6.41 × 10${}^{-2}$	1.23 × 10${}^{-5}$	2.64 × 10${}^{-2}$
2	2.63 × 10${}^{-2}$	2.81 × 10${}^{-2}$	3.25 × 10${}^{-2}$	1.09 × 10${}^{-2}$	4.37 × 10${}^{-2}$	5.95 × 10${}^{-2}$	2.76 × 10${}^{-2}$
3	1.38 × 10${}^{-1}$	2.20 × 10${}^{-1}$	8.59 × 10${}^{-2}$	6.26 × 10${}^{-2}$	1.53 × 10${}^{-1}$	2.61 × 10${}^{-1}$	1.56 × 10${}^{-1}$
4	1.58 × 10${}^{-1}$	1.71 × 10${}^{-2}$	6.35 × 10${}^{-2}$	9.72 × 10${}^{-2}$	6.20 × 10${}^{-1}$	4.68 × 10${}^{-1}$	1.01 × 10${}^{-1}$
5	1.36 × 10${}^{-2}$	5.24 × 10${}^{-2}$	3.27 × 10${}^{-2}$	3.78 × 10${}^{-2}$	4.72 × 10${}^{-5}$	9.11 × 10${}^{-3}$	1.55 × 10${}^{-2}$
	$C/C^{\infty }$	$N/N^{\infty }$	$\mu_{1}/\mu_{1}^{\infty }$	$\mu_{2}/\mu_{2}^{\infty }$	$H/H^{\infty }$	$I_{\gamma }/I_{\gamma }^{\infty }$	$G_{\beta }/G_{\beta }^{\infty }$
1	4.64 × 10${}^{-2}$	1.55 × 10${}^{-2}$	4.97 × 10${}^{-1}$	5.12 × 10${}^{-1}$	1.47	3.89	2.55 × 10${}^{-1}$
2	2.38 × 10${}^{-2}$	1.46 × 10${}^{-3}$	7.58 × 10${}^{-1}$	1.79 × 10${}^{-1}$	6.04 × 10${}^{-1}$	9.38 × 10${}^{-1}$	5.57 × 10${}^{-1}$
3	1.83 × 10${}^{-1}$	0	7.51 × 10${}^{-1}$	7.14 × 10${}^{-1}$	1.03	4.90 × 10${}^{-1}$	3.87
4	1.22 × 10${}^{-1}$	0	4.14 × 10${}^{-1}$	5.77 × 10	1.46 × 10	0	2.56 × 10
5	2.69 × 10${}^{-2}$	7.03 × 10${}^{-3}$	4.59 × 10${}^{-1}$	2.30	1.18	4.08 × 10${}^{-1}$	3.46 × 10${}^{-1}$

**Table 3:** Drug dosages in mg.

	Fluorouracil	Leucovorin	Irinotecan
median	770	725	300
min	598	75	208
number of cycles	12	12	12
cycle length	14	14	14

**Table 4:** Number of patients in the validation data.

	Tumor Free	With Tumor
Early follow up day	23	8
Last follow up day	24	12

## Data Availability

The TCGA data of COAD project underlying this article are available at https://portal.gdc.cancer.gov and https://xenabrowser.net/datapages/ (accessed on 30 March 2021) [113]. Codes are available at our GitHub page https://github.com/ShahriyariLab/Data-driven-mathematical-model-of-FOLFIRI-treatment-for-colon-cancer (accessed on 30 March 2021).

## Abbreviations

The following abbreviations are used in this manuscript:

COAD	Colon adenocarcinoma
CRC	Colorectal cancer
HMGB1	High mobility group box 1
DAMP	damage-associated molecular pattern
TAM	Tumor-associated macrophages
ODE	Ordinary differential equation
TYMS	thymidylate synthase
TCD	Total cell density
TIC	Total immune cell

## Appendix A. ODE System & Non-Dimensionalization

The entire ODE system, which has been modeled in this paper is given below. The activation and proliferation rates are denoted by λ, while death and degradation rates are denoted by δ. Changes and additions to the system modeled in [48] are highlighted in bold.

(A1) $$\begin{array}{c} \begin{array}{cc} \frac{d[T_{N}]}{dt}= & A_{T_{N}}-\left(\lambda_{T_{h}D}\left[D\right]+\lambda_{T_{h}D5\mathrm{fu}}\left[5\mathrm{FU}\right]\left[D\right]+\lambda_{T_{h}M}\left[M\right]+\lambda_{T_{h}\mu_{1}}\left[\mu_{1}\right]\right)\left[T_{N}\right]\\ & -\left(\lambda_{T_{C}T_{h}}\left[T_{h}\right]+\lambda_{T_{C}D}\left[D\right]+\lambda_{T_{C}D5\mathrm{fu}}\left[5\mathrm{FU}\right]\left[D\right]\right)\left[T_{N}\right]\\ & -\left(\lambda_{T_{r}T_{h}}\left[T_{h}\right]+\lambda_{T_{r}\mu_{2}}\left[\mu_{2}\right]+\lambda_{T_{r}G_{\beta }}\left[G_{\beta }\right]\right)\left[T_{N}\right]-\delta_{T_{N}}\left[T_{N}\right] \end{array} \end{array}$$

(A2) $$\begin{array}{c} \begin{array}{cc} \frac{d[T_{h}]}{dt}= & \left(\lambda_{T_{h}D}\left[D\right]+\lambda_{T_{h}D5\mathrm{fu}}\left[5\mathrm{FU}\right]\left[D\right]+\lambda_{T_{h}M}\left[M\right]+\lambda_{T_{h}\mu_{1}}\left[\mu_{1}\right]\right)\left[T_{N}\right]\\ - & \left(\delta_{T_{h}\mu_{2}}\left[\mu_{2}\right]+\delta_{T_{h}T_{r}}\left[T_{r}\right]+\delta_{T_{h}}\right)\left[T_{h}\right] \end{array} \end{array}$$

(A3) $$\begin{array}{c} \begin{array}{cc} \frac{d[T_{C}]}{dt}= & \left(\lambda_{T_{C}T_{h}}\left[T_{h}\right]+\lambda_{T_{C}D}\left[D\right]+\lambda_{T_{C}D5\mathrm{fu}}\left[5\mathrm{FU}\right]\left[D\right]\right)\left[T_{N}\right]\\ - & \left(\delta_{T_{C}\mu_{2}}\left[\mu_{2}\right]+\delta_{T_{C}T_{r}}\left[T_{r}\right]+\delta_{T_{C}}\right)\left[T_{C}\right] \end{array} \end{array}$$

(A4) $$\begin{array}{cc} \frac{d[T_{r}]}{dt}= & \left(\lambda_{T_{r}T_{h}}\left[T_{h}\right]+\lambda_{T_{r}\mu_{2}}\left[\mu_{2}\right]+\lambda_{T_{r}G_{\beta }}\left[G_{\beta }\right]\right)\left[T_{N}\right]-\left(\delta_{T_{r}\mu_{1}}\left[\mu_{1}\right]+\delta_{T_{r}}+\delta_{T_{r}\mathrm{Ir}}\left[\mathrm{Ir}\right]\right)\left[T_{r}\right] \end{array}$$

(A5) $$\begin{array}{cc} \frac{d\left[D_{N}\right]}{dt}= & A_{D_{N}}-\left(\lambda_{DH}\left[H\right]+\lambda_{DC}\left[C\right]\right)\left[D_{N}\right]-\left(\delta_{DH}\left[H\right]+\delta_{D}\right)\left[D_{N}\right] \end{array}$$

(A6) $$\begin{array}{cc} \frac{d\left[D\right]}{dt}= & \left(\lambda_{DH}\left[H\right]+\lambda_{DC}\left[C\right]\right)\left[D_{N}\right]-\left(\delta_{DH}\left[H\right]+\delta_{DC}\left[C\right]+\delta_{D}\right)\left[D\right] \end{array}$$

(A7) $$\begin{array}{cc} \frac{d\left[M\right]}{dt}= & \left(\lambda_{M\mu_{2}}\left[\mu_{2}\right]+\lambda_{MI_{\gamma }}\left[I_{\gamma }\right]+\lambda_{MT_{h}}\left[T_{h}\right]\right)\left(M_{0}-\left[M\right]\right)-\delta_{M}\left[M\right] \end{array}$$

(A8) $$\begin{array}{c} \begin{array}{cc} \frac{d[C]}{dt}= & \left(\lambda_{C}+\lambda_{C\mu_{1}}\left[\mu_{1}\right]\right)\left[C\right]\left(1-\frac{\left[C\right]}{C_{0}}\right)-(\delta_{CG_{\beta }}\left[G_{\beta }\right]+\delta_{CI_{\gamma }}\left[I_{\gamma }\right]+\delta_{CT_{C}}\left[T_{C}\right]+\delta_{C}\\ & +\delta_{C5\mathrm{fu}}\left[5\mathrm{FU}\right]+\delta_{C5\mathrm{fu}I_{\gamma }}\left[5\mathrm{FU}\right]\left[I_{\gamma }\right]-\delta_{5\mathrm{fu}M}\left[5\mathrm{FU}\right]\left[M\right]\\ + & \delta_{C\mathrm{LV}5\mathrm{fu}}\left[5\mathrm{FU}\right]\left[\mathrm{LV}\right]+\delta_{C\mathrm{Ir}}\left[\mathrm{Ir}\right])\left[C\right] \end{array} \end{array}$$

(A9) $$\begin{array}{c} \begin{array}{cc} \frac{d[N]}{dt}= & \alpha_{NC}{(\delta }_{CG_{\beta }}\left[G_{\beta }\right]+\delta_{CI_{\gamma }}\left[I_{\gamma }\right]+\delta_{CT_{C}}\left[T_{C}\right]+\delta_{C}\\ & +\delta_{C5\mathrm{fu}}\left[5\mathrm{FU}\right]+\delta_{C5\mathrm{fu}I_{\gamma }}\left[5\mathrm{FU}\right]\left[I_{\gamma }\right]-\delta_{5\mathrm{fu}M}\left[5\mathrm{FU}\right]\left[M\right]\\ + & \delta_{C\mathrm{LV}5\mathrm{fu}}\left[5\mathrm{FU}\right]\left[\mathrm{LV}\right]+\delta_{C\mathrm{Ir}}\left[\mathrm{Ir}\right])\left[C\right]-\delta_{N}\left[N\right] \end{array} \end{array}$$

(A10) $$\begin{array}{cc} \frac{d\left[H\right]}{dt}= & \lambda_{HN}\left[N\right]+\lambda_{HM}\left[M\right]+\lambda_{HT_{h}}\left[T_{h}\right]+\lambda_{HT_{C}}\left[T_{C}\right]+\lambda_{HT_{r}}\left[T_{r}\right]-\delta_{H}\left[H\right] \end{array}$$

(A11) $$\begin{array}{cc} \frac{d\left[\mu_{1}\right]}{dt}= & \lambda_{\mu_{1}T_{h}}\left[T_{h}\right]+\lambda_{\mu_{1}M}\left[M\right]+\lambda_{\mu_{1}D}\left[D\right]-\delta_{\mu_{1}}\left[\mu_{1}\right] \end{array}$$

(A12) $$\begin{array}{cc} \frac{d\left[\mu_{2}\right]}{dt}= & \lambda_{\mu_{2}M}\left[M\right]+\lambda_{\mu_{2}D}\left[D\right]+\lambda_{\mu_{2}T_{r}}\left[T_{r}\right]-\delta_{\mu_{2}}\left[\mu_{2}\right] \end{array}$$

(A13) $$\begin{array}{cc} \frac{d\left[I_{\gamma }\right]}{dt}= & \lambda_{I_{\gamma }T_{h}}\left[T_{h}\right]+\lambda_{I_{\gamma }T_{C}}\left[T_{C}\right]+\lambda_{I_{\gamma }M}\left[M\right]-\delta_{I_{\gamma }}\left[I_{\gamma }\right] \end{array}$$

(A14) $$\begin{array}{cc} \frac{d\left[G_{\beta }\right]}{dt}= & \lambda_{G_{\beta }M}\left[M\right]+\lambda_{G_{\beta }T_{r}}\left[T_{r}\right]-\delta_{G_{\beta }}\left[G_{\beta }\right] \end{array}$$

(A15) $$\begin{array}{c} \begin{array}{cc} \frac{d[5\mathrm{FU}]}{\mathrm{dt}}= & A_{\mathrm{inj}}^{5\mathrm{fu}}\left(t\right)-\alpha_{5\mathrm{fu}}(\delta_{C5\mathrm{fu}}\left[5\mathrm{FU}\right]+\delta_{C5\mathrm{fu}I_{\gamma }}\left[5\mathrm{FU}\right]\left[I_{\gamma }\right]-\delta_{5\mathrm{fu}M}\left[5\mathrm{FU}\right]\left[M\right]\\ \; & +\delta_{C\mathrm{LV}5\mathrm{fu}}\left[5\mathrm{FU}\right]\left[\mathrm{LV}\right])\left[C\right]-\delta_{5\mathrm{fu}D}\left[5\mathrm{FU}\right]\left[D\right]-\delta_{5\mathrm{fu}}\left[5\mathrm{FU}\right] \end{array} \end{array}$$

(A16) $$\begin{array}{cc} \frac{d[\mathrm{Ir}]}{\mathrm{dt}}= & A_{\mathrm{inj}}^{\mathrm{Ir}}\left(t\right)-\alpha_{\mathrm{Ir}C}\;\delta_{C\mathrm{Ir}}\left[C\right]\left[\mathrm{Ir}\right]-\alpha_{\mathrm{Ir}T_{r}}\;\delta_{T_{r}\mathrm{Ir}}\left[\mathrm{Ir}\right]\left[T_{r}\right]-\delta_{\mathrm{Ir}}\left[\mathrm{Ir}\right] \end{array}$$

(A17) $$\begin{array}{cc} \frac{d[\mathrm{LV}]}{\mathrm{dt}}= & A_{\mathrm{inj}}^{\mathrm{LV}}\left(t\right)-\delta_{\mathrm{LV}}\left[\mathrm{LV}\right]-\alpha_{\mathrm{LV}}\;\delta_{C\mathrm{LV}5\mathrm{fu}}\left[C\right]\left[5\mathrm{FU}\right]\left[\mathrm{LV}\right] \end{array}$$

We introduce non-dimensional variables,

$$\begin{array}{cccccc} \bar{5FU}= & \frac{\delta_{5fu}\left[5FU\right]}{{\langle A_{inj}^{5fu}\rangle }_{daily\;median}} & \bar{Ir}= & \frac{\delta_{Ir}\left[Ir\right]}{{\langle A_{inj}^{Ir}\rangle }_{daily\;median}}\; & \bar{LV}= & \frac{\delta_{LV}\left[LV\right]}{{\langle A_{inj}^{LV}\rangle }_{daily\;median}}, \end{array}$$

then we have the following non-dimensional equations (only the new and altered ones have been included, the rest of non-dimensionalized equations are exactly the same as the ones provided in [48]):

(A18) $$\begin{array}{c} \begin{array}{cc} \frac{d[\bar{5\mathrm{FU}}]}{\mathrm{dt}}= & {\bar{A}}_{\mathrm{inj}}^{5\mathrm{fu}}\left(t\right)-{\bar{\alpha }}_{5\mathrm{fu}}({\bar{\delta }}_{C5\mathrm{fu}}\left[\bar{5\mathrm{FU}}\right]+{\bar{\delta }}_{C5\mathrm{fu}I_{\gamma }}\left[\bar{5\mathrm{FU}}\right]\left[\bar{I_{\gamma }}\right]-{\bar{\delta }}_{5\mathrm{fu}M}\left[\bar{5\mathrm{FU}}\right]\left[\bar{M}\right]\\ & +{\bar{\delta }}_{C\mathrm{LV}5\mathrm{fu}}\left[\bar{5\mathrm{FU}}\right]\left[\bar{\mathrm{LV}}\right])\left[\bar{C}\right]-{\bar{\delta }}_{5\mathrm{fu}D}\left[\bar{5\mathrm{FU}}\right]\left[\bar{D}\right]-\delta_{5\mathrm{fu}}\left[\bar{5\mathrm{FU}}\right] \end{array} \end{array}$$

(A19) $$\begin{array}{cc} \frac{d[\bar{\mathrm{Ir}}]}{\mathrm{dt}}= & {\bar{A}}_{\mathrm{inj}}^{\mathrm{Ir}}\left(t\right)-{\bar{\alpha }}_{\mathrm{Ir}C}\;{\bar{\delta }}_{C\mathrm{Ir}}\left[\bar{C}\right]\left[\bar{\mathrm{Ir}}\right]-{\bar{\alpha }}_{\mathrm{Ir}T_{r}}\;{\bar{\delta }}_{T_{r}\mathrm{Ir}}\left[\bar{\mathrm{Ir}}\right]\left[{\bar{T}}_{r}\right]-\delta_{\mathrm{Ir}}\left[\bar{\mathrm{Ir}}\right] \end{array}$$

(A20) $$\begin{array}{cc} \frac{d[\bar{\mathrm{LV}}]}{\mathrm{dt}}= & {\bar{A}}_{\mathrm{inj}}^{\mathrm{LV}}\left(t\right)-\delta_{\mathrm{LV}}\left[\bar{\mathrm{LV}}\right]-{\bar{\alpha }}_{\mathrm{LV}}\;{\bar{\delta }}_{C\mathrm{LV}5\mathrm{fu}}\left[\bar{C}\right]\left[\bar{5\mathrm{FU}}\right]\left[\bar{\mathrm{LV}}\right] \end{array}$$

(A21) $$\begin{array}{c} \begin{array}{cc} \frac{d[\bar{T_{r}}]}{dt}= & \left({\bar{\lambda }}_{T_{r}T_{h}}\left[{\bar{T}}_{h}\right]+{\bar{\lambda }}_{T_{r}\mu_{2}}\left[\mu_{2}\right]+{\bar{\lambda }}_{T_{r}G_{\beta }}\left[{\bar{G}}_{\beta }\right]\right)\left[{\bar{T}}_{N}\right]\\ & -\left({\bar{\delta }}_{T_{r}\mu_{1}}\left[{\bar{\mu }}_{1}\right]+\delta_{T_{r}}+{\bar{\delta }}_{T_{r}\mathrm{Ir}}\left[\bar{\mathrm{Ir}}\right]\right)\left[{\bar{T}}_{r}\right] \end{array} \end{array}$$

(A22) $$\begin{array}{c} \begin{array}{cc} \frac{d[\bar{T_{h}}]}{dt}= & \left({\bar{\lambda }}_{T_{h}D}\left[\bar{D}\right]+{\bar{\lambda }}_{T_{h}D5\mathrm{fu}}\left[\bar{5\mathrm{FU}}\right]\left[\bar{D}\right]+{\bar{\lambda }}_{T_{h}M}\left[\bar{M}\right]+{\bar{\lambda }}_{T_{h}\mu_{1}}\left[{\bar{\mu }}_{1}\right]\right)\left[\bar{T_{N}}\right]\\ & -\left({\bar{\delta }}_{T_{h}\mu_{2}}\left[{\bar{\mu }}_{2}\right]+{\bar{\delta }}_{T_{h}T_{r}}\left[{\bar{T}}_{r}\right]+\delta_{T_{h}}\right)\left[\bar{T_{h}}\right] \end{array} \end{array}$$

(A23) $$\begin{array}{c} \begin{array}{cc} \frac{d[\bar{T_{C}}]}{dt}= & \left({\bar{\lambda }}_{T_{C}T_{h}}\left[\bar{T_{h}}\right]+{\bar{\lambda }}_{T_{C}D}\left[\bar{D}\right]+{\bar{\lambda }}_{T_{C}D5\mathrm{fu}}\left[\bar{5\mathrm{FU}}\right]\left[\bar{D}\right]\right)\left[\bar{T_{N}}\right]\\ & -\left({\bar{\delta }}_{T_{C}\mu_{2}}\left[{\bar{\mu }}_{2}\right]+{\bar{\delta }}_{T_{C}T_{r}}\left[{\bar{T}}_{r}\right]+\delta_{T_{C}}\right)\left[\bar{T_{C}}\right] \end{array} \end{array}$$

(A24) $$\begin{array}{c} \begin{array}{cc} \frac{d[\bar{T_{N}}]}{dt}= & {\bar{A}}_{T_{N}}-\alpha_{T_{N}T_{h}}\left({\bar{\lambda }}_{T_{h}D}\left[\bar{D}\right]+{\bar{\lambda }}_{T_{h}D5\mathrm{fu}}\left[\bar{5\mathrm{FU}}\right]\left[\bar{D}\right]+{\bar{\lambda }}_{T_{h}M}\left[\bar{M}\right]+{\bar{\lambda }}_{T_{h}\mu_{1}}\left[{\bar{\mu }}_{1}\right]\right)\left[\bar{T_{N}}\right]\\ & -\alpha_{T_{N}T_{C}}\left({\bar{\lambda }}_{T_{C}T_{h}}\left[\bar{T_{h}}\right]+{\bar{\lambda }}_{T_{C}D}\left[\bar{D}\right]+{\bar{\lambda }}_{T_{C}D5\mathrm{fu}}\left[\bar{5\mathrm{FU}}\right]\left[\bar{D}\right]\right)\left[\bar{T_{N}}\right]\\ & -\alpha_{T_{N}T_{r}}\left({\bar{\lambda }}_{T_{r}T_{h}}\left[\bar{T_{h}}\right]+{\bar{\lambda }}_{T_{r}\mu_{2}}\left[{\bar{\mu }}_{2}\right]+{\bar{\lambda }}_{T_{r}G_{\beta }}\left[{\bar{G}}_{\beta }\right]\right)\left[\bar{T_{N}}\right]-\delta_{T_{N}}\left[\bar{T_{N}}\right] \end{array} \end{array}$$

(A25) $$\begin{array}{c} \begin{array}{cc} \frac{d[C]}{dt}= & \left(\lambda_{C}+{\bar{\lambda }}_{C\mu_{1}}\left[{\bar{\mu }}_{1}\right]\right)\left[\bar{C}\right]\left(1-\frac{\left[C\right]}{C_{0}}\right)-({\bar{\delta }}_{CG_{\beta }}\left[{\bar{G}}_{\beta }\right]+{\bar{\delta }}_{CI_{\gamma }}\left[{\bar{I}}_{\gamma }\right]+{\bar{\delta }}_{CT_{C}}\left[{\bar{T}}_{C}\right]+\delta_{C}\\ & +{\bar{\delta }}_{C5\mathrm{fu}}\left[\bar{5\mathrm{FU}}\right]+{\bar{\delta }}_{C5\mathrm{fu}I_{\gamma }}\left[\bar{5\mathrm{FU}}\right]\left[{\bar{I}}_{\gamma }\right]-{\bar{\delta }}_{5\mathrm{fu}M}\left[\bar{5\mathrm{FU}}\right]\left[\bar{M}\right]\\ & +{\bar{\delta }}_{C\mathrm{LV}5\mathrm{fu}}\left[\bar{5\mathrm{FU}}\right]\left[\bar{\mathrm{LV}}\right]+{\bar{\delta }}_{C\mathrm{Ir}}\left[\bar{\mathrm{Ir}}\right])\left[\bar{C}\right] \end{array} \end{array}$$

where the newly introduced non-dimensional parameters are:

$$\begin{array}{cccc} {\bar{A}}_{inj}^{5fu} & =\frac{A_{inj}^{5fu}\delta_{5fu}}{{\langle A_{inj}^{5fu}\rangle }_{daily\;median}} & {\bar{\alpha }}_{5fu} & =\frac{\alpha_{5fu}C^{\infty }\delta_{5fu}}{{\langle A_{inj}^{5fu}\rangle }_{daily\;median}}\\ {\bar{\lambda }}_{T_{h}D5fu} & =\frac{\lambda_{T_{h}D5fu}D^{\infty }T_{N}^{\infty }{\langle A_{inj}^{5fu}\rangle }_{daily\;median}}{\delta_{5fu}T_{h}^{\infty }} & {\bar{\delta }}_{5fuD} & =\delta_{5fuD}D^{\infty }\\ {\bar{\lambda }}_{T_{C}D5fu} & =\frac{\lambda_{T_{C}D5fu}D^{\infty }T_{N}^{\infty }{\langle A_{inj}^{5fu}\rangle }_{daily\;median}}{\delta_{5fu}T_{C}^{\infty }}\; & {\bar{\delta }}_{C5fu} & =\frac{\delta_{C5fu}{\langle A_{inj}^{5fu}\rangle }_{daily\;median}}{\delta_{5fu}}\\ {\bar{\delta }}_{C5fuI_{\gamma }} & =\frac{\delta_{C5fuI_{\gamma }}I_{\gamma }^{\infty }{\langle A_{inj}^{5fu}\rangle }_{daily\;median}}{\delta_{5fu}} & {\bar{\delta }}_{5fuM} & =\frac{\delta_{5fuM}M^{\infty }{\langle A_{inj}^{5fu}\rangle }_{daily\;median}}{\delta_{5fu}}\\ {\bar{\delta }}_{CLV5fu} & =\delta_{CLV5fu}\cdot \frac{\left[\bar{5FU}\right]{\langle A_{inj}^{5fu}\rangle }_{daily\;median}}{\delta_{5fu}}\cdot \frac{\left[\bar{LV}\right]{\langle A_{inj}^{LV}\rangle }_{daily\;median}}{\delta_{LV}} & {\bar{A}}_{inj}^{LV} & =\frac{A_{inj}^{LV}\;\delta_{LV}}{{\langle A_{inj}^{LV}\rangle }_{daily\;median}}\\ {\bar{\alpha }}_{LV} & =\frac{\alpha_{LV}C^{\infty }\delta_{LV}}{{\langle A_{inj}^{LV}\rangle }_{daily\;median}}\; & {\bar{\delta }}_{CIr} & =\frac{\delta_{CIr}{\langle A_{inj}^{Ir}\rangle }_{daily\;median}}{\delta_{Ir}}\\ {\bar{A}}_{inj}^{Ir} & =\frac{A_{inj}^{Ir}\;\delta_{Ir}}{{\langle A_{inj}^{Ir}\rangle }_{daily\;median}} & {\bar{\delta }}_{T_{r}Ir} & =\frac{\delta_{T_{r}Ir}{\langle A_{inj}^{Ir}\rangle }_{daily\;median}}{\delta_{Ir}}\\ {\bar{\alpha }}_{IrC} & =\frac{\alpha_{IrC}C^{\infty }\delta_{Ir}}{{\langle A_{inj}^{Ir}\rangle }_{daily\;median}} & {\bar{\alpha }}_{IrT_{r}} & =\frac{\alpha_{IrT_{r}}T_{r}^{\infty }\delta_{Ir}}{{\langle A_{inj}^{Ir}\rangle }_{daily\;median}} \end{array}$$

Note that $A_{inj}\left(t\right)$ represents the infusion step function and ${\langle A_{inj}\rangle }_{daily\;min/median}$ is the corresponding drug minimum or median administered dose based on patients’ data as given in Table 3.

For the natural decay rates of the drugs, we use the same formula from [48] by using their respective terminal/elimination half-lives as reported in available studies.

From available studies, we know that the terminal half-life of 5-FU is in between 8 and 20 min [135]. Therefore, using the average half-life as 14 min, we have

$$\delta_{5fu}=\frac{\ln2}{t_{1/2}}=\frac{\ln2}{14\;\mathrm{mins}}=71.3\;{\mathrm{day}}^{-1}.$$

Since Leucovorin is a mixture of the two isomers [6R]-5-formyl-tetrahydrofolate and [6S]-5-formyl-tetrahydrofolate, both with very different half-lives, to determine its half-life, we use [136], which reports it as 5.7 h and the terminal half-life as given by [137] for the total folates and metabolites of 7.59 h. Therefore, taking the average half-life as 6.5 h (approx. 5–8 h) we have

$$\delta_{LV}=\frac{\ln2}{6.5\;\mathrm{hrs}}=2.56\;{\mathrm{day}}^{-1}$$

And the reported terminal half-life of Irinotecan ranges between 5 and 18 h [103,138,139], giving an average half-life of 11.5 h. Therefore,

$$\delta_{Ir}=\frac{\ln2}{11.5\;\mathrm{hrs}}=1.45\;{\mathrm{day}}^{-1}$$

The median dosages are used for the parameters ${\langle A_{inj}\rangle }_{daily\;median}$ corresponding to each drug by dividing them by the average cycle length to obtain the daily dose:

$${\langle A_{inj}\rangle }_{daily\;median}^{5fu}=median\;dosage\;per\;cycle\;per\;day=\frac{770}{14}$$

$${\langle A_{inj}\rangle }_{daily\;median}^{Ir}=median\;dosage\;per\;cycle\;per\;day=\frac{300}{14}$$

$${\langle A_{inj}\rangle }_{daily\;median}^{LV}=median\;dosage\;per\;cycle\;per\;day=\frac{725}{14}$$

Similarly, the minimum daily dose per cycle from patients’ data for each corresponding drug is the minimum dose divided by the average cycle length:

$${\langle A_{inj}\rangle }_{daily\;min}^{5fu}=min.\;dosage\;per\;cycle\;per\;day=\frac{598}{14}$$

$${\langle A_{inj}\rangle }_{daily\;min}^{Ir}=min.\;dosage\;per\;cycle\;per\;day=\frac{208}{14}$$

$${\langle A_{inj}\rangle }_{daily\;min}^{LV}=min.\;dosage\;per\;cycle\;per\;day=\frac{75}{14}$$

We used the following assumptions for the parameter values (all in dimensional form). Values for αs have been derived from their efficiency, either from available studies or by appropriate assumptions. Note that maximum values and steady state values of the variables are taken from [48].

$$\begin{array}{cc} \delta_{5fuI_{\gamma }}\cdot \frac{I_{\gamma }^{max}}{I_{\gamma }^{\infty }}= & \delta_{C5fu}\cdot \frac{3}{2}\\ \delta_{5fuM}\cdot \frac{M^{max}}{M^{\infty }}= & {\bar{\delta }}_{5fuD}\cdot \frac{D^{max}}{D^{\infty }}=\frac{1}{2}\cdot \frac{\delta_{C}}{{\langle A_{inj}^{5fu}\rangle }_{daily\;min}}\\ \delta_{C5fu}\cdot {\langle A_{inj}^{5fu}\rangle }_{daily\;min}= & 10^{2}\cdot \delta_{C}\\ \lambda_{T_{h}D5fu}\cdot {\langle A_{inj}^{5fu}\rangle }_{daily\;min}= & \frac{1}{2}\cdot {\bar{\lambda }}_{T_{h}D}\\ \lambda_{T_{C}D5fu}\cdot {\langle A_{inj}^{5fu}\rangle }_{daily\;min}= & \frac{1}{2}\cdot {\bar{\lambda }}_{T_{C}D}\\ \alpha_{5fu}= & \mathrm{derived}\;\mathrm{from}\;\mathrm{efficiency} \end{array}$$

It is assumed that about 20% of 5-FU is effectively used to kill cancer cells (as reported above [28,107,108]).

$$\begin{array}{cc} \delta_{C5fuLV}= & 0.1\cdot \frac{\delta_{C5fu}}{{\langle A_{inj}^{LV}\rangle }_{daily\;min}}\\ \alpha_{LV}= & \mathrm{derived}\;\mathrm{from}\;\mathrm{efficiency} \end{array}$$

assuming 5% efficiency of killing cancer cells for Leucovorin.

$$\begin{array}{cc} \delta_{CIr}= & 0.5\cdot \delta_{C5fu}\cdot \frac{{\langle A_{inj}^{5fu}\rangle }_{daily\;min}}{{\langle A_{inj}^{Ir}\rangle }_{daily\;min}} \end{array}$$

$$\begin{array}{cc} \delta_{IrT_{r}}= & \delta_{CIr} \end{array}$$

$$\begin{array}{cc} \alpha_{IrT_{r}}= & \alpha_{IrC} \end{array}$$

$$\begin{array}{cc} \alpha_{IrC}=\; & \mathrm{derived}\;\mathrm{from}\;\mathrm{efficiency} \end{array}$$

assuming 40% efficiency of killing cancer cells for Irinotecan, since at least 60% of the drug is known to be eliminated from the body without being used [103].

In non-dimensional form, the parameter assumptions are as follows:

$$\begin{array}{cccc} {\bar{\lambda }}_{T_{h}D5fu}= & \frac{{\bar{\lambda }}_{T_{h}D}{\langle A_{inj}^{5fu}\rangle }_{daily\;median}}{2\cdot \delta_{5fu}{\langle A_{inj}^{5fu}\rangle }_{daily\;min}}\; & {\bar{\lambda }}_{T_{C}D5fu}= & \frac{{\bar{\lambda }}_{T_{C}D}{\langle A_{inj}^{5fu}\rangle }_{daily\;median}}{2\cdot \delta_{5fu}{\langle A_{inj}^{5fu}\rangle }_{daily\;min}}\\ {\bar{\delta }}_{C5fuI_{\gamma }}= & \frac{3\cdot {\bar{\delta }}_{C5fu}\cdot I_{\gamma }^{\infty }}{2\cdot I_{\gamma }^{max}}\; & {\bar{\delta }}_{5fuM}= & \frac{\delta_{C}\cdot M^{\infty }\cdot {\langle A_{inj}^{5fu}\rangle }_{daily\;median}}{2\cdot \delta_{5fu}\cdot M^{max}\cdot {\langle A_{inj}^{5fu}\rangle }_{daily\;min}}\\ {\bar{\delta }}_{5fuD}= & \frac{\delta_{C}\cdot D^{\infty }\cdot {\langle A_{inj}^{5fu}\rangle }_{daily\;median}}{2\cdot \delta_{5fu}\cdot D^{max}\cdot {\langle A_{inj}^{5fu}\rangle }_{daily\;min}}\; & {\bar{\delta }}_{C5fu}= & 10^{2}\cdot \frac{{\langle A_{inj}^{5fu}\rangle }_{daily\;median}}{{\langle A_{inj}^{5fu}\rangle }_{daily\;min}}\cdot \frac{\delta_{C}}{\delta_{5fu}}\\ {\bar{\delta }}_{CIr}= & \frac{1}{2}\cdot \frac{{\langle A_{inj}^{IR}\rangle }_{daily\;median}}{{\langle A_{inj}^{IR}\rangle }_{daily\;min}}\frac{\delta_{5fu}{\bar{\delta }}_{C5fu}}{\delta_{IR}}\frac{{\langle A_{inj}^{IR}\rangle }_{daily\;min}}{{\langle A_{inj}^{IR}\rangle }_{daily\;median}}\; & {\bar{\delta }}_{T_{r}Ir}= & {\bar{\delta }}_{CIr}\\ {\bar{\delta }}_{C5fuLV}= & 0.1\cdot \frac{{\langle A_{inj}^{5fu}\rangle }_{daily\;median}}{{\langle A_{inj}^{LV}\rangle }_{daily\;min}}\cdot \frac{{\bar{\delta }}_{C5fu}}{\delta_{LV}}\; & {\bar{\alpha }}_{LV}= & \frac{LV_{eff}}{1-LV_{eff}}\cdot \frac{\delta_{LV}}{{\bar{\delta }}_{C5fuLV}\left(1-5fu_{eff}\right)}\\ {\bar{\alpha }}_{IrC}= & \frac{IR_{eff}}{1-IR_{eff}}\frac{\delta_{Ir}}{{\bar{\delta }}_{CIr}}\; & {\bar{\alpha }}_{IrT_{r}}= & \frac{IR_{eff}}{1-IR_{eff}}\frac{\delta_{Ir}}{{\bar{\delta }}_{T_{r}Ir}}\\ {\bar{\alpha }}_{5fu}= & \frac{\left(\frac{5FU_{eff}}{1-5FU_{eff}}\right)\delta_{5fu}-{\bar{\delta }}_{5fuD}}{{\bar{\delta }}_{C5fu}+{\bar{\delta }}_{C5fuI_{\gamma }}-\delta_{5fuM}+\delta_{C5fuLV}\cdot \left(1-LV_{eff}\right)} \end{array}$$
